# The entangled triplet pair state in acene and heteroacene materials

**DOI:** 10.1038/ncomms15953

**Published:** 2017-07-12

**Authors:** Chaw Keong Yong, Andrew J. Musser, Sam L. Bayliss, Steven Lukman, Hiroyuki Tamura, Olga Bubnova, Rawad K. Hallani, Aurélie Meneau, Roland Resel, Munetaka Maruyama, Shu Hotta, Laura M. Herz, David Beljonne, John E. Anthony, Jenny Clark, Henning Sirringhaus

**Affiliations:** 1 Cavendish Laboratory, Optoelectronics Group, University of Cambridge, Madingley Road, J.J. Thomson Avenue, Cambridge CB3 0HE, UK; 2 Department of Physics, University of California, Berkeley, California 94720, USA; 3 Department of Physics and Astronomy, The University of Sheffield, Hicks Buildling, Hounsfield Road, Sheffield S3 7RH, UK; 4 Department of Chemical System Engineering, The University of Tokyo, 7-3-1, Hongo, Bunkyo-ku, Tokyo 113-8656, Japan; 5 Department of Chemistry, University of Kentucky, Lexington, Kentucky 40506-0055, USA; 6 Institute of Solid State Physics, Graz University of Technology, Petersgasse 16, 8010 Graz, Austria; 7 Faculty of Materials Science and Engineering, Kyoto Institute of Technology, Matsugasaki, Sakyo-ku, Kyoto 606-8585, Japan; 8 Clarendon Laboratory, Department of Physics, University of Oxford, Parks Road, Oxford OX1 3PU, UK; 9 Laboratory for Chemistry of Novel Materials, University of Mons, Place du Parc 20, B-7000 Mons, Belgium

## Abstract

Entanglement of states is one of the most surprising and counter-intuitive consequences of quantum mechanics, with potent applications in cryptography and computing. In organic materials, one particularly significant manifestation is the spin-entangled triplet-pair state, which mediates the spin-conserving fission of one spin-0 singlet exciton into two spin-1 triplet excitons. Despite long theoretical and experimental exploration, the nature of the triplet-pair state and inter-triplet interactions have proved elusive. Here we use a range of organic semiconductors that undergo singlet exciton fission to reveal the photophysical properties of entangled triplet-pair states. We find that the triplet pair is bound with respect to free triplets with an energy that is largely material independent (∼30 meV). During its lifetime, the component triplets behave cooperatively as a singlet and emit light through a Herzberg–Teller-type mechanism, resulting in vibronically structured photoluminescence. In photovoltaic blends, charge transfer can occur from the bound triplet pairs with >100% photon-to-charge conversion efficiency.

## Abstract (web summary)

Singlet fission in organic semiconductors can generate triplet exciton pairs that are crucial to the charge generation in a photovoltaic process, whilst their nature remains elusive. Here, Yong *et al*. show that the immediate triplet pair is bound and emissive in a range of acene and heteroacene materials.

The entangled pair of triplet excitons ^1^(TT) is thought to be essential to the rapid, spin-allowed formation of two triplets from a single absorbed photon^1^,^2^,^3^. Its overall singlet character may allow for surprisingly fast, long-distance triplet exciton transport following singlet fission^4^. The very same state also mediates the reverse of singlet fission: triplet–triplet annihilation (TTA), where two triplets fuse to form an emissive singlet, a process exploited for biomedical imaging^5^,^6^, high-density secure data storage^7^, incoherent energy up-conversion^8^ and high-efficiency blue organic light-emitting diodes^9^. Beyond applications of singlet fission and TTA, a clearer understanding of the ^1^(TT) state will shed light on the nature of the interaction between triplet excitons and may open the way to new physics involving entangled bosons^10^,^11^. Despite significant theoretical effort^10^,^11^,^12^,^13^,^14^,^15^,^16^,^17^,^18^,^19^,^20^, few experiments have succeeded in directly probing the ^1^(TT) state in any system^21^,^22^,^23^,^24^,^25^, particularly in solid films relevant to eventual singlet fission devices. We are aware of a recent study of the ^1^(TT) state in solid films of 6,13-Bis(triisopropylsilylethynyl) (TIPS)-tetracene, which crystallizes into a structure unique among singlet fission materials^26^. The nature of this state and its role in singlet fission in the wider range of acene and heteroacene singlet-fission materials are not clear, nor its importance in the physics of singlet fission-based solar cells. During the review process, several other reports have emerged describing properties of triplet-pair states, including dynamic equilibrium with S_1_ (ref. 27), the formation of quintet-coupled triplet pairs^28^,^29^, ultrafast formation in a strongly exothermic system^30^ and theoretical re-evaluations of triplet-pair interactions^31^,^32^,^33^. These works highlight the ongoing debate about the nature of ^1^(TT). Of those that discuss energetics, all consider ^1^(TT) to be higher in energy than T_1_+T_1_. Here we demonstrate this is incorrect and show that ^1^(TT) is bound with respect to free triplets.

Here we probe the ^1^(TT) state experimentally and theoretically in a range of acene and heteroacene materials (Fig. 1a). We demonstrate the generality of the presence of a bound ^1^(TT) state as the immediate product of singlet fission. The processes of formation and decay of ^1^(TT) are shown in Fig. 1b, a schematic inspired by Tayebjee *et al*.^34^ and references therein 13,35. Formation of ^1^(TT) is temperature-independent in polycrystalline films and is thought to occur via a combination of intra- and intermolecular motion from the photoexcited singlet^12^,^13^,^14^,^21^,^34^,^36^,^37^. Once formed, ^1^(TT) can undergo one of three detectable decay processes as follows: (i) form free (unbound) triplets, a process aided by disorder at low temperatures and thermally activated above 50 K; (ii) emit (red arrows) by way of symmetry-breaking distortions enabling intensity borrowing from nearby bright states; and (iii) with sufficient energy, ‘back transfer’ to S_1_ leading to delayed fluorescence. We also infer that a significant proportion of ^1^(TT) states undergo non-radiative decay, presumably directly to the ground state. In photovoltaic blends, we find that charge transfer from this bound, entangled triplet pair is as efficient as that from free triplets giving photon-to-charge conversion quantum efficiencies of >100%.

## Results

### Emissive triplet-pair state

**Figure 1:**
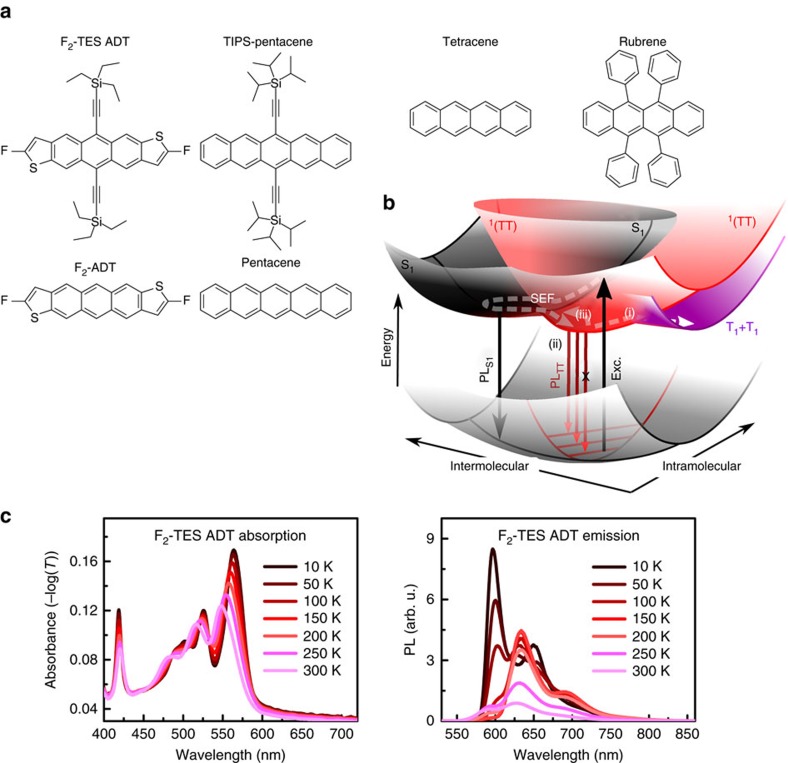
Singlet exciton fission in polyacenes. (**a**) Molecules investigated in this study. (**b**) Schematic potential energy surface denoting key photophysical processes (dashed arrows) in singlet fission materials. Photoexcitation (Exc.) is followed by singlet (black) relaxation along inter- and intramolecular coordinates, resulting in the observed Stokes shift (PL_S1_). Further relaxation into the bound, spin-coherent triplet pair state ^1^(TT) (red) constitutes singlet exciton fission (SEF). Three detectable decay processes are possible from ^1^(TT). (i) Thermally activated dissociation into free triplets (purple), which is aided by disorder/grain boundaries and exhibits a typical activation energy of 20–40 meV. (ii) Direct ‘delayed’ emission from ^1^(TT) (PL_TT_) through Herzberg–Teller intensity borrowing. As a consequence of this mechanism, the 0-0 transition is suppressed. (iii) Thermally activated back-transfer into the singlet manifold. This process results in delayed fluorescence (PL_S1_), and is suppressed in tetracene and F_2_-TES ADT at ≤ 200 K, indicating the presence of a slight energy barrier. (**c**) Temperature-dependent absorption and PL spectra of F_2_-TES ADT films.

Figure 1a shows the chemical structures of the molecules used. All undergo singlet fission with time constants ranging from sub-100 fs in pentacene and TIPS-pentacene to 50–300 ps in 2,8-difluoro-5,11-bis(triethylsilylethynyl)anthradithiophene (F_2_-TES ADT), rubrene and tetracene^15^,^38^,^39^,^40^. The time-integrated, temperature-dependent photoluminescence (PL) spectra of F_2_-TES ADT are shown in Fig. 1c. Similar to tetracene films, described in refs 34, 41, 42, 43, emission at temperatures between 200 and 90 K is dominated by a new peak, red-shifted from the 0–0 emission peak. Below 90 K, an enhanced 0–0 peak once again dominates the spectrum. Given the similarity in temperature-dependent emission and, as we demonstrate below, singlet fission dynamics in F_2_-TES ADT and tetracene, we suggest a similar origin for the red-shifted emissive species. We propose in the following that this emission arises from a bound, entangled triplet pair, ^1^(TT), the immediate product of singlet fission.

**Figure 2:**
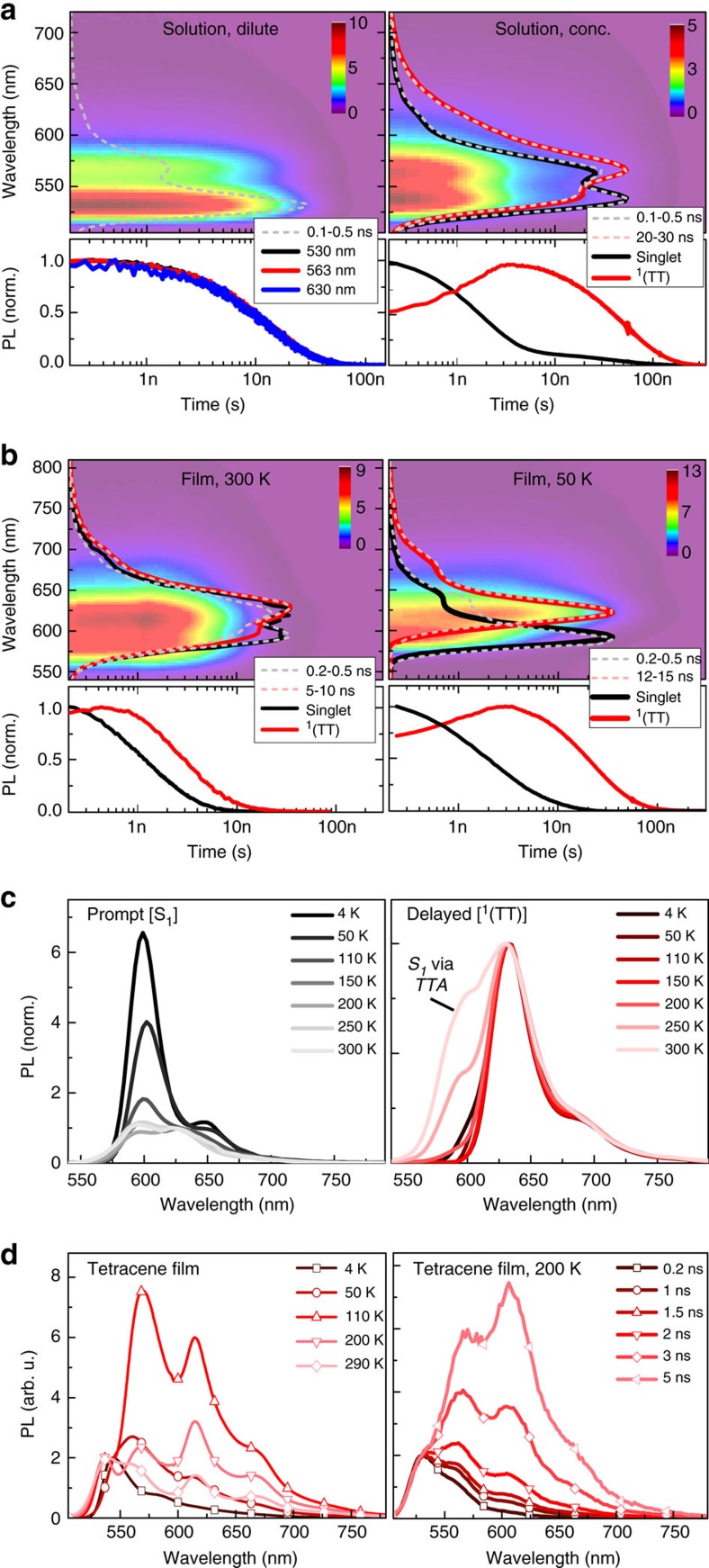
^1^(TT) emission. F_2_-TES ADT time-resolved PL spectral maps for (**a**) solutions and (**b**) thin films. Prompt and delayed spectral components (solid lines) were extracted as described in the main text. Dashed lines are normalized spectral cuts from raw PL at the indicated time delays. Associated population kinetics (bottom) reveal sequential conversion from prompt to delayed emission. (**c**) Prompt and delayed F_2_-TES ADT film spectra extracted over the full temperature range, attributed to S_1_ and ^1^(TT), respectively. The delayed PL broadening at >200 K reveals thermal repopulation of S_1_ from ^1^(TT) via TTA, process (iii) in Fig. 1b. (**d**) Normalized PL spectra of a tetracene film as a function of temperature in steady-state conditions (left) and time after excitation at 200 K (right).

To better understand the origin of the PL spectral shifts, we performed time-resolved measurements in F_2_-TES ADT solutions (Fig. 2a) and thin films (Fig. 2b). We chose to focus on F_2_-TES ADT as, unlike tetracene, F_2_-TES ADT shows no significant structural phase transition in the thin films (Supplementary Fig. 4), no evidence of ‘excimer’ emission^43^,^44^ and strong triplet features in transient absorption (TA) spectra. In dilute solution only singlet states emit, with a lifetime of 12 ns. Concentrated solutions and thin films, however, show evidence of multiple emitting species. To separate spectral features with correlated decay kinetics we use a spectral decomposition method based on a genetic algorithm^45^ (see the Methods section for more details). The PL maps are best modelled as a sum of two independent species (solid lines) both in concentrated solution and thin films. The same procedure was applied to data taken at other temperatures (Supplementary Fig. 5), yielding clearly delineated prompt and delayed PL spectra (Fig. 2c).

In concentrated solution, the singlet converts into a red-shifted species maintaining vibronic structure. Likewise, in solid films decomposition reveals correlation between prompt and delayed emitter kinetics: the delayed species grows at the expense of the prompt. Although the ‘prompt’ emitter lifetime remains essentially unchanged over the temperature range, the ‘delayed’ emitter lifetime increases substantially with decreasing temperature. Conversely, the prompt emission spectrum changes dramatically with temperature. The enhancement of the 0–0 peak, a property common to materials such as tetracene, which show super-radiance at low temperature^46^, allows assignment to the singlet exciton. The delayed red-shifted emission spectrum, which dominates F_2_-TES ADT film emission at intermediate temperatures, is remarkably constant below 200 K and can be assigned to ^1^(TT), see below. At higher temperatures, the increased intensity in the delayed component at 585 nm, co-incident with the singlet 0–0 emission, suggests thermally activated delayed fluorescence with the same lifetime as ^1^(TT) (process (iii) in Fig. 1b).

### Spin-entanglement of ^1^(TT) observed by quantum beating

**Figure 3:**
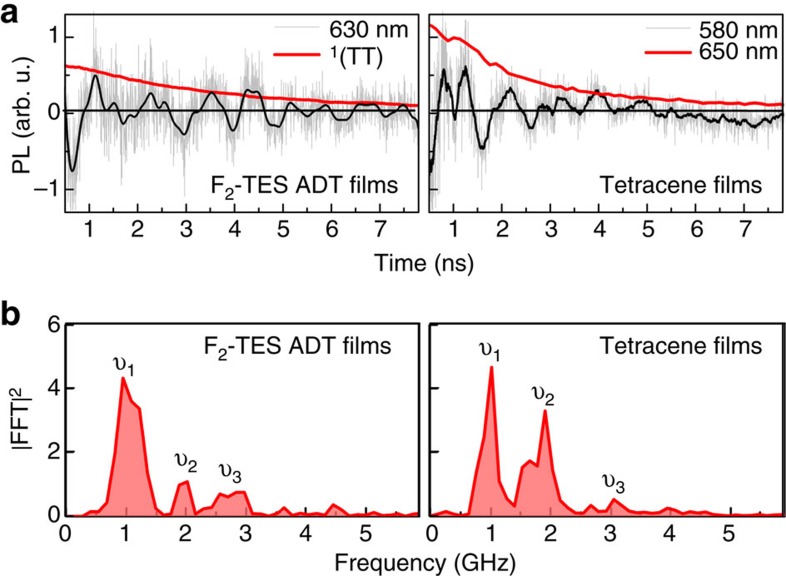
PL quantum beating from spin-entangled ^1^(TT). (**a**) PL decay of F_2_-TES ADT and tetracene films following femtosecond excitation at 300 K, after subtraction of multi-exponential envelope. Resulting slow oscillations (grey: raw, black: smoothed) decay in agreement with extracted ^1^(TT) kinetics in F_2_-TES ADT and red-emitting feature in tetracene (red). (**b**) Fourier transform of the oscillations, revealing quantum beats at 1.05, 1.96, 2.88±0.1 GHz (F_2_-TES ADT) and 0.99, 1.91 and 3.06±0.1 GHz (tetracene).

Interestingly, following femtosecond excitation at 300 K we observed temporal oscillations of the PL, as shown in Fig. 3a. Similar to tetracene^47^,^48^ (right panel), the amplitude reduces gradually within an 8 ns window and follows the extracted delayed emitter kinetics. The Fourier transform reveals the existence of three beat frequencies (Fig. 3b) at 1.1, 2.0 and 2.9±0.1 GHz that are strikingly similar to those in tetracene (1.0, 1.9 and 3.1±0.1 GHz, respectively), as discussed in Supplementary Note 1. These quantum beats have never been reported in other materials than tetracene, and are a manifestation of the spin properties of triplet pairs following singlet fission^47^,^48^. Briefly, in the simplest two-electron two-hole picture of singlet fission, there are 16 possible combinations of 4 spins: 2 singlets, 9 triplets and 5 quintets. Singlet fission entails spin-allowed conversion between the two singlet states, one of which is a superposition of triplet-pair states of the form 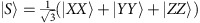 in the zero-field basis (see Supplementary Notes 1 and 2 for details including magnetic resonance characterization (Supplementary Fig. 6) and details relating to quantum beating (Supplementary Fig. 7)). |*S*〉 is not an energy eigenstate of the system and |*XX*〉, |*YY*〉 and |*ZZ*〉 acquire relative phases, evolving in time with frequencies governed by their energy spacing. As the state periodically approaches its initial composition (that is, ^1^(TT)), there is significant enhancement of the rate to re-form the original, fluorescent singlet through TTA if this is energetically allowed. The observed PL quantum beats thus reveal modulation of the radiative TTA probability due to spin conservation. The oscillation amplitude follows the extracted delayed PL kinetics in F_2_-TES ADT and tetracene, clearly linking the delayed emission to the ^1^(TT) state generated by singlet fission. The beats’ persistence over the ^1^(TT) lifetime indicates that spin coherence is maintained over at least that timescale, and that ^1^(TT) is thus an entangled triplet-pair state.

### ^1^(TT) emission through Herzberg–Teller symmetry breaking

It is unexpected for this doubly excited state (^1^(TT)) to emit, especially with the well-defined vibronic progression seen in Fig. 2c, right panel. To probe the origin of this behaviour we have performed *ab initio* (CASSCF(4,4)) excited-state calculations on *π*-stacked dimers in the crystal geometry (details in the Methods section below and Table 1). Mixing with charge transfer and singlet states stabilizes ^1^(TT) relative to the singlet and non-interacting triplet pairs. Importantly, the bright singlet contribution to the adiabatic ^1^(TT) state vanishes at the equilibrium geometry^16^,^20^. ^1^(TT) instead mixes only with a dark singlet, with emission thus forbidden. Geometric distortion activates slight mixing with the bright state, resulting in finite but low radiative rate (that is, ∼40 × lower than that of the initial singlet state in F_2_-TES ADT at 50 K). Nonetheless, the ^1^(TT) emission can dominate the spectrum at intermediate temperatures due to its long lifetime. This mechanism is equivalent to Herzberg–Teller coupling^49^, in which a symmetry-forbidden dark state can couple to a vibration of the bright-state symmetry and thereby obtain the correct symmetry for mixing or intensity borrowing from nearby bright states.

**Figure 4:**
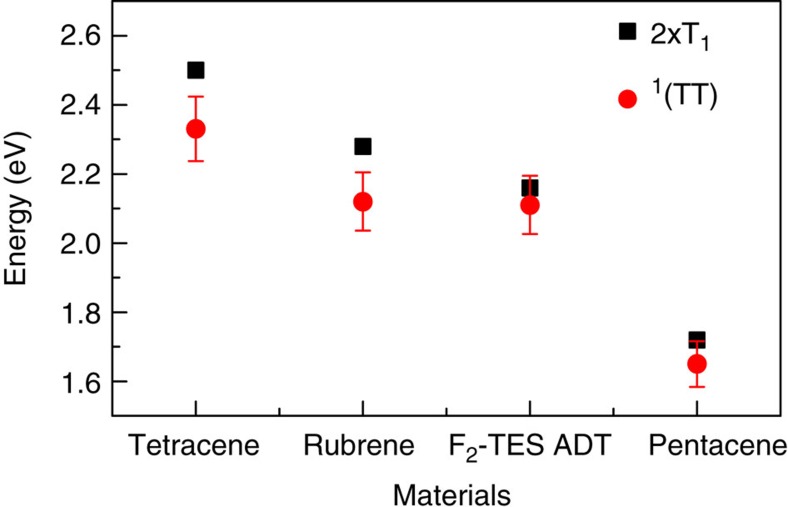
^1^(TT) energies. ^1^(TT) and 2 × T_1_ energies for materials studied here: ^1^(TT) energy extracted from PL spectra, see Supplementary Fig. 1, T_1_ from phosphorescence measurements (F_2_-TES ADT, rubrene Supplementary Figs 2, 3) and literature^61^,^73^. Error bars reflect uncertainty in fitting the ^1^(TT) vibronic progression.

We propose that this mechanism explains the vibronically structured red-shifted PL seen in many acenes in the literature. As discussed above, tetracene demonstrates very similar temperature-dependent PL to F_2_-TES ADT (Fig. 2d, Supplementary Note 3, Supplementary Figs 8 and 9, and refs 34, 41, 42, 43), with a red-shifted delayed component^34^,^42^ that decays with the same lifetime as the quantum beating (Fig. 3a). Even on much longer timescales, structured ^1^(TT) emission may be preferentially regenerated by annihilation from ‘trapped’ triplets due to its lower electronic energy^34^,^50^. Rubrene (Supplementary Fig. 10) likewise shows evidence of a temperature-dependent long-lived PL feature, somewhat obscured by the similarity of the S_1_ and ^1^(TT) energies; even pentacene has been shown to demonstrate similarly shaped emission in films^51^ and crystals^52^. Under Herzberg–Teller coupling the 0–0 peak is suppressed^53^; thus, we assign the most prominent feature in the ^1^(TT) spectrum to the 0–1 peak, akin to emission from the ‘triplet-pair’-like^54^ S_1_ state in polyenes^55^. Following this model, we determine the energy of the ^1^(TT) 0–0 peak from the spacing between observed 0–1 and 0–2 peaks (Supplementary Fig. 1). We find that the energy extracted from PL scales with the expected 2 × T_1_ energy from independent measurements of the triplet energy, with an offset which could account for the binding energy of the triplets in ^1^(TT). This is shown in Fig. 4.

This model has critical implications for singlet fission energetics in ‘slow’ tetracene-like materials. In F_2_-TES ADT and similar materials, ^1^(TT) and S_1_ are roughly isoenergetic. Indeed, the delayed spectra in Fig. 2c reveal temperature-dependent broadening consistent with delayed singlet re-formation, which is turned off <200 K. The implicit small barrier to singlet re-formation can only be reconciled with the observed ^1^(TT) PL spectrum through the Herzberg–Teller model. The ability of ^1^(TT) to emit and the spin-conservation considerations above suggest that quantum beating could likewise be anticipated in the direct ^1^(TT) emission, suggesting a need for spectrally resolved quantum beating studies.

### Identification of ^1^(TT) in TA spectroscopy

**Figure 5:**
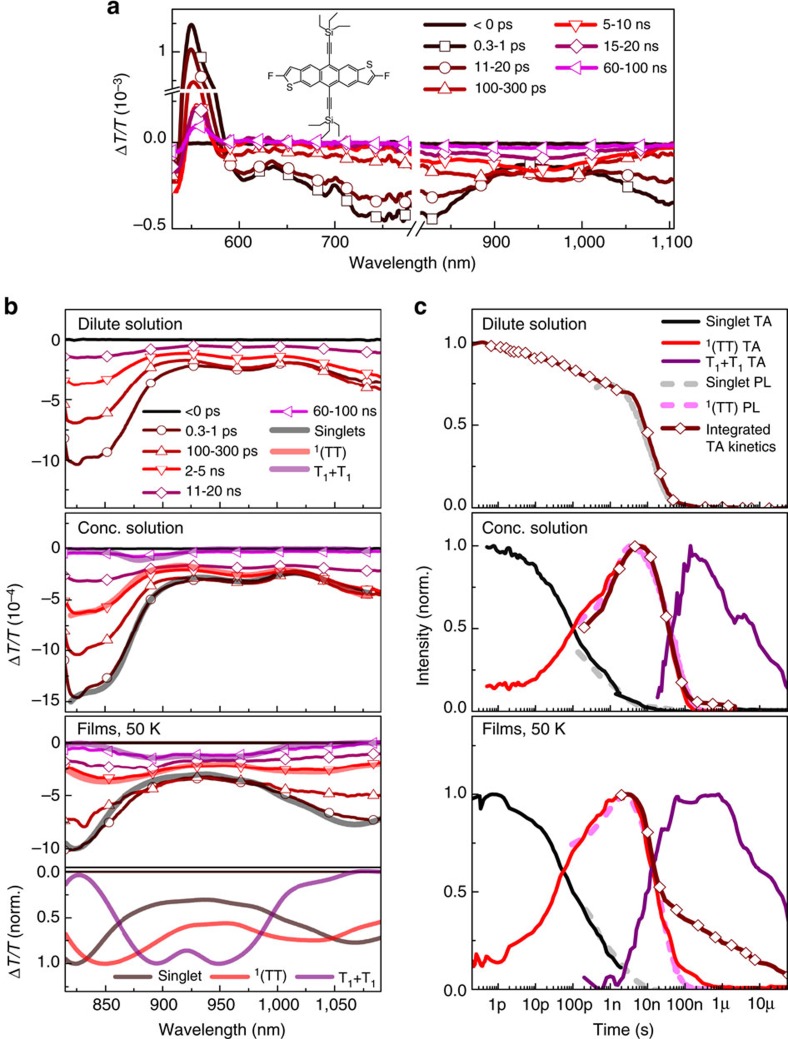
^1^(TT) absorption in F_2_-TES ADT. (**a**) TA spectra of F_2_-TES ADT films at 300 K, averaged over the indicated delay ranges. The initial singlet state (peaks 750–800 nm and >1,100 nm) converts directly into triplet excitons (peak centred at 970 nm) over ∼300 ps, through singlet fission. (**b**) TA spectra of F_2_-TES ADT dilute solution (top, 0.08 mM), concentrated solution (middle, 32 mM) and thin film measured at 50 K (bottom). Extracted spectra (thick lines) closely follow the raw data at different time delays, revealing the presence of substantial triplet population (T_1_+T_1_) in concentrated solutions and thin film, as well as a distinct intermediate state ^1^(TT). Extracted spectra of the three species in the film at 50 K are normalized in the bottom panel for clarity. (**c**) Normalized population kinetics of excited state species extracted from TA maps, exhibiting a sequential progression: singlet (black), ^1^(TT) (red) and free triplets (purple). Also plotted are the integrated NIR TA signal (brown, diamonds) and kinetics extracted from the separate transient PL experiment (dashed lines). The agreement of PL and TA population kinetics confirms the same species are observed in both experiments.

For further insight into the formation and decay of ^1^(TT) we used TA spectroscopy. Figure 5a shows the TA spectra of F_2_-TES ADT films collected at 300 K and we can immediately distinguish two primary species: the initially formed singlet exciton and a subsequent long-lived photo-induced absorption (PIA) band centred at 970 nm. To aid assignment of this feature, we examine F_2_-TES ADT solutions at several concentrations^21^,^36^ (Fig. 5b,c). For simplicity, we focus on the probe range 800–1,100 nm, where all states of interest have distinct PIA features (full characterization in Supplementary Figs 5, 11-13). In dilute solutions (Fig. 5b,c top), we observe ∼12 ns singlet decay consistent with measured PL (Fig. 2a). Concentrated solutions (32 mM, Fig. 5b,c middle) exhibit faster singlet decay and spectral evolution over 100s of nanoseconds to yield long-lived triplets (T_1_+T_1_; sensitization^56^ in Supplementary Fig. 11). Between singlet and triplet, we observe an intermediate, which decays with the same kinetics (red trace) as the delayed ^1^(TT) emission (pink dashed trace), enabling assignment to ^1^(TT). The last panels in Fig. 5b,c reveal a similar progression in thin films at 50 K (Supplementary Fig. 13 for other temperatures), demonstrating that ^1^(TT) acts as a distinct singlet fission intermediate even in the solid state. We expect the ^1^(TT) PIA absorption cross-section to be similar to that of two free triplets. Therefore, the reduction in signal from 3–100 ns in the raw TA kinetic (pink trace in Fig. 5c) suggests significant decay without forming free triplets. The total PL quantum yield of 10% at 50 K indicates that this decay is largely non-radiative.

**Figure 6:**
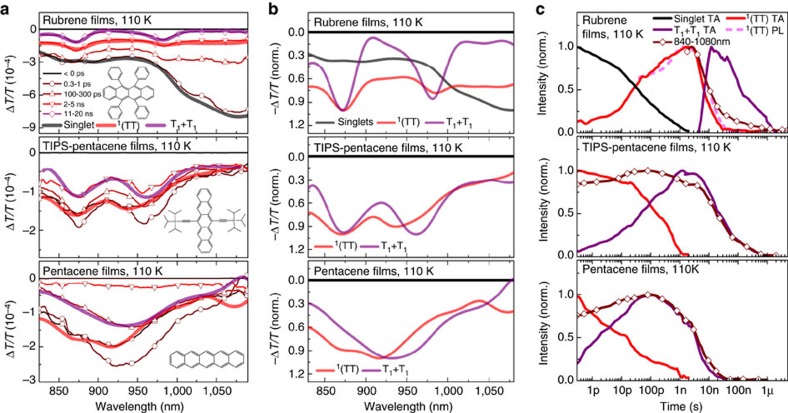
^1^(TT)-mediated singlet fission in polyacenes. (**a**) TA spectra of rubrene, TIPS-pentacene and pentacene thin films measured at low temperature, averaged over the indicated delay ranges (thin lines). Spectral decomposition analysis (thick lines) provides spectra of singlet (black), ^1^(TT) (red) and free triplet (purple). Note the rate of singlet fission in TIPS-pentacene and pentacene is too fast to clearly isolate singlet spectral features. In all materials, the conversion from ^1^(TT) to free triplets can be identified from peak shifts in the PIA. (**b**) Extracted singlet, ^1^(TT) and free triplet spectra from (**a**) normalized for clarity. (**c**) Normalized population kinetics of excited state species extracted from TA maps, along with integrated raw TA signal (brown, diamonds) and extracted PL kinetics, where applicable (dashed). All materials exhibit the same sequential model.

We observe the same progression in rubrene, TIPS-pentacene and pentacene (Fig. 6), confirming the presence of the ^1^(TT) intermediate in all three (see also Supplementary Figs 14–18). In rubrene, these features match the red-shifted delayed PL kinetics (Supplementary Fig. 10). No PL was detected in TIPS-pentacene and pentacene films. We performed similar measurements on polycrystalline tetracene films (Supplementary Fig. 19) but, as discussed in Supplementary Note 3, find that these are difficult due to small triplet absorption cross-sections^43^,^57^,^58^,^59^, coupled with the need for low excitation density to avoid exciton–exciton annihilation^60^. In the other materials (Supplementary Fig. 20), ^1^(TT) decay was completely independent of intensity, indicating that any recombination is geminate. By contrast, free triplets exhibit fluence-dependent recombination. Interestingly, our results show that S_1_ decay and ^1^(TT) growth are largely temperature-independent (Fig. 5 and Supplementary Figs 13 and 14), even in rubrene^39^.

### Stabilization of ^1^(TT) versus S_1_ and 2 × T_1_

**Figure 7:**
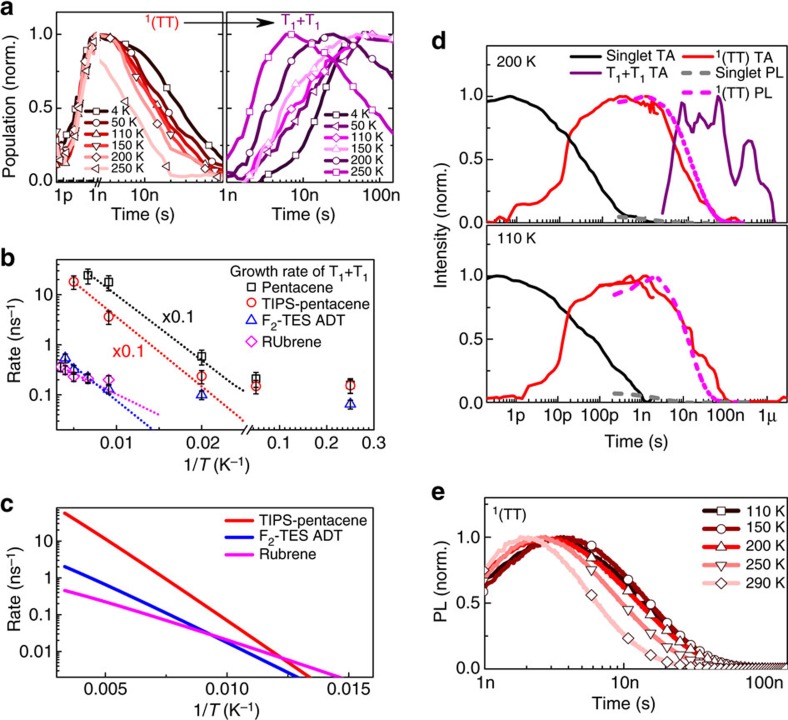
^1^(TT) decay through activated triplet hopping. (**a**) Kinetics of ^1^(TT) and free triplet species in F_2_-TES ADT films, extracted from TA measurements over the temperature range 4–250 K and revealing temperature-dependent conversion. Kinetics presented on separate time axes for clarity. (**b**) Rate of ^1^(TT) decay as a function of temperature. Error bars are uncertainty in the exponential fitting of ^1^(TT) decay. Dashed lines are mono-exponential fits to the high-temperature data. Although the overall rate of ^1^(TT) separation varies widely, the temperature dependence is strikingly similar across the series (activation energy ∼20–40 meV). Data for pentacene-based materials scaled by 0.1 × for clarity. (**c**) Marcus–Levich–Jortner rate model of ^1^(TT) separation for three *π*-stacked systems. The strong variation in rate is attributed to the triplet-transfer coupling, while the temperature dependence is primarily governed by the enthalpy of ^1^(TT) separation. (**d**) Normalized population kinetics of excited-state species extracted from TA measurements (solid) and PL measurements (dashed) of F_2_-ADT single crystal at 200 and 110 K (see also Supplementary Fig. 21). As in thin films, sequential conversion from S_1_ to emissive ^1^(TT) to free triplets is observed, but ^1^(TT) separation is fully suppressed at low temperature. (**e**) Normalized, temperature-dependent decay kinetics of ^1^(TT) in F_2_-ADT single crystal. At low temperature where no free triplets are formed, the decay converges to the intrinsic ^1^(TT) lifetime.

A temperature dependence does, however, appear in the subsequent process, in which the bound triplet pair separates into free triplet excitons (Fig. 5 and Supplementary Fig. 13). It is noteworthy that this is even observed in TIPS-pentacene and pentacene (Supplementary Note 4 and Supplementary Figs 16 and 18), where the overall process of singlet fission is exothermic (Supplementary Fig. 21 and ref. 61). In Fig. 7a we plot ^1^(TT) and free triplet population dynamics for F_2_-TES ADT films from 4–250 K. Data for other molecules can be found in Supplementary Fig. 20 (F_2_ ADT single crystal), Supplementary Fig. 13 (rubrene), Supplementary Fig. 15 (TIPS-pentacene) and Supplementary Fig. 17 (pentacene). From an Arrhenius fit to the corresponding rates of free triplet formation at elevated temperature, we obtain a general phenomenological activation barrier ∼20–40 meV (Fig. 7b), although the underlying rates vary by nearly two orders of magnitude. These results demonstrate that ^1^(TT) is stabilized with respect not only to the initial singlet but also to two free triplet excitons, confirming its bound nature^21^. We consider that this behaviour is general to the acenes and is likely to be a common property of all singlet fission materials, recalling the well-known stabilization of the triplet-pair 2A_g_ state in polyenes relative to two free triplets^54^,^56^,^62^.

To better understand this binding and thermally activated decay, we have analysed conversion from ^1^(TT) into free triplets based on Marcus–Levich–Jortner rate theory parameterized against *ab initio* calculations (Table 2 and Fig. 7c, full details in the Methods section). We considered the *π*-stacked crystal geometries of F_2_-TES ADT, TIPS-pentacene and rubrene, in which separation is one dimensional. The enthalpy change Δ*H* for separation was determined using trimer models in which molecules adopted either the fully relaxed triplet geometry (*T*) or the ground-state geometry (*G*). Bound and separated triplet pairs then correspond to TTG and TGT configurations, respectively. The free energy stabilization going from TGT to TTG completely dominates the activation energy Δ*E*. The stabilization energies are overestimated by these calculations, probably due to the presence of polymorphism and the assumed single crystal geometry. For instance, we calculate that increasing the *π*-stack distance in TIPS-pentacene by 0.1 Å decreases the Δ*H* by ∼30%. Nonetheless, the predicted trends in the Δ*E* values match well with measurements.

Moreover, the magnitudes of Δ*H* (50–100 meV) correlate well with the admixture of singlet and charge-transfer configurations (Table 1), indicating that this mixing is a primary driver of ^1^(TT) stabilization, although orbital delocalization also contributes. The electronic coupling for intermolecular triplet transfer *V*_T−T_ was estimated using *π*-stacked dimer models and agrees well with previous studies^38^.

As shown in Fig. 7d, the resulting rate equation yields a strong temperature dependence for ^1^(TT) separation, in good agreement with experimental results given the simple model. Importantly, we reproduce the large rate difference between TIPS-pentacene and F_2_-TES ADT, and the smaller difference between F_2_-TES ADT and rubrene. These differences cannot be attributed to Δ*H* (comparable for all three) and instead follow *V*_T−T_. Thus, the underlying rate for ^1^(TT) separation is governed by the intrinsic triplet mobility. At the same time, we note that the overall activation energy is dominated by Δ*H* rather than the inter- or intramolecular reorganization energies. We can thus conclude that the measured high-temperature activation energies reflect the energy stabilization of bound versus free triplet pairs, and that triplet-pair separation can be described in terms of triplet hopping from the bound state. The most significant discrepancy between theory and experiment is at low temperature: the model predicts vanishingly small ^1^(TT) separation, whereas we measure very little change in the rate from ∼50 K to lower temperatures. We propose that structural and/or energetic disorder, for instance as would be found at grain boundaries, accounts for the persistence of low-barrier free triplet formation.

To verify this, we have performed similar detailed TA and PL measurements on single crystals of difluorinated anthradithiophene (F_2_-ADT, Fig. 7d,e and Supplementary Fig. 20). We observed similar singlet and ^1^(TT) emission, with clear temperature dependence of the ^1^(TT) lifetime above 200 K but little temperature dependence below. In TA, we found free triplet formation at ≥200 K but observed no free triplets at lower temperature. These results support our model for the role of disorder: in the absence of structural or energetic disorder, the ^1^(TT) state must be separated through thermal energy alone, leading to complete suppression of the process at low temperature.

### Concerted two-electron transfer from ^1^(TT) in solar cells

**Figure 8:**
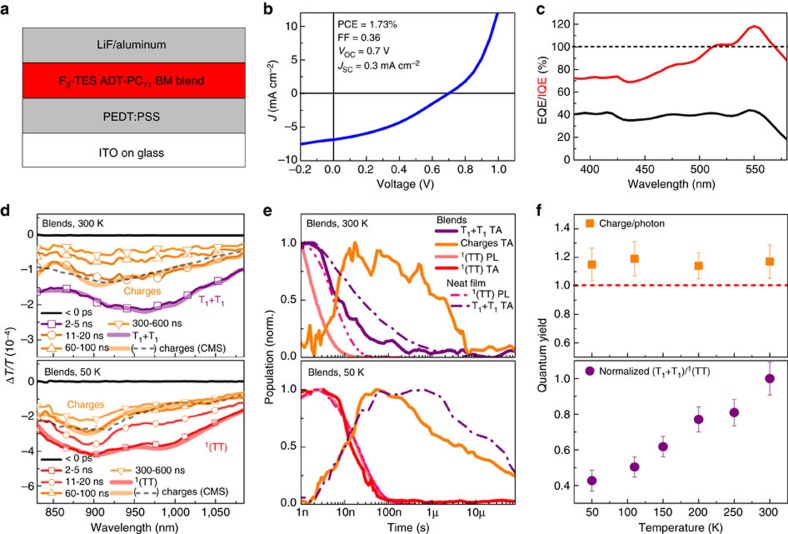
^1^(TT) in photovoltaic blends. (**a**) Schematic photovoltaic (PV) device structure. (**b**) Typical IV curve for F_2_-TES ADT:PC_71_BM blend PV with corresponding power conversion efficiency (PCE), fill-factor (FF), open-circuit voltage (*V*_oc_) and short-circuit current (*J*_sc_) as indicated. (**c**) Despite the low PCE of 1.7%, a plot of external and internal quantum efficiency (external quantum efficiency (EQE) and internal quantum efficiency (IQE) respectively) shows >100% IQE in the region where F_2_-TES ADT absorbs. (**d**) TA spectra of bulk-heterojunction films of F_2_-TES ADT and PC_71_BM in 4:1 molar ratio, at 300 K (top) and 50 K (bottom) and averaged over the indicated delay ranges (thin lines). Thick lines: species extracted from spectral decomposition. The F_2_-TES ADT hole polaron (radical cation or ‘charge’) spectra extracted through spectral decomposition closely match those obtained with charge-modulation spectroscopy (CMS, dashed). (**e**) Triplet and charge population kinetics extracted from data in (**d**) and Fig. 6. (neat films, dash-dotted) and equivalent transient PL maps. Shortening of both the ^1^(TT) PL lifetime and the free triplet lifetime at 300 K suggest both species contribute to charge formation. At lower temperatures, no free triplets are detected prior to charge transfer at the interface. The kinetics of interfacial charge transfer closely match those of triplet-pair separation in neat films (dash-dotted). (**f**) Top panel: photon-to-charge conversion yield determined from TA measurements (errors from uncertainty in amplitude of TA signal and charge cross-section) of blend films reveals negligible temperature dependence. In contrast, the yield of free triplets in neat films (bottom panel, error bars reflect noise in raw TA signal), plotted as a ratio of T_1_+T_1_ to ^1^(TT) from TA measurements and normalized to the 300 K value, varies with temperature.

The low rate for ^1^(TT) separation in F_2_-TES ADT, tetracene^47^ and rubrene suggests that this state may play a significant role in fission-sensitized solar cells, unlike in pentacene-based devices where the extrapolated room-temperature ^1^(TT) lifetime is only ∼200 fs (ref. 22). To elucidate the behaviour of ^1^(TT) in photovoltaic blend films and devices, we have studied bulk-heterojunction films of F_2_-TES ADT and [6,6]-phenyl C71 butyric acid methyl ester (PC_71_BM) in 4:1 molar ratio, in which we expect significant demixing. The device structure is shown schematically in Fig. 8a with electrical characteristics in Fig. 8b,c. We focus here on two representative temperatures: 300 and 50 K, where the primary long-lived species in F_2_-TES ADT should either be free or bound spin-entangled triplets, respectively (for other temperatures, see Supplementary Fig. 22). As noted above, the yield of free triplets relative to ^1^(TT) drops as the temperature is lowered (Fig. 8f).

Figure 8d,e shows that at 300 K free triplets are formed, revealing that a significant proportion of F_2_-TES ADT molecules must be far from the fullerene interface. Spectral decomposition (Fig. 8d) reveals a third species, identified as a hole polaron (radical cation) in F_2_-TES ADT from comparison with charge-modulation spectroscopy (dashed line in Fig. 8d and Supplementary Fig. 23). Charge-modulation spectroscopy is used to determine the optical spectra and absorption cross-section of hole polarons in the solid-state (see Supplementary Note 5 for details). From the strength of the hole polaron contribution to the TA signal we can then directly extract the charge population formed (see Supplementary Note 5 and Supplementary Table 1), resulting in a photon-to-charge quantum yield of ∼120%. Equivalent device characterization in Fig. 8b,c shows that, although the power conversion efficiency of the device is low (1.7%), the peak internal quantum efficiency approached 120% in the spectral range of F_2_-TES ADT (Fig. 8c) in excellent agreement with the TA results.

The 300 K extracted kinetics shown in Fig. 8e reveal that these charges form at the expense of both bound and free triplet excitons, resulting in a reduction of both the ^1^(TT) PL and free triplet TA lifetimes. There is thus a competition between two modes of ^1^(TT) decay: diffusion to a PC_71_BM interface with direct charge generation, and thermally activated separation into free triplets, which in turn diffuse to the interface to form charges.

At 50 K, the contribution of ^1^(TT) to charge formation can be isolated, as free triplet formation is strongly suppressed. Indeed, we find that the relative yield of free triplets is >50% lower at 50 K than at 300 K (Fig. 8f). In the TA dynamics, following formation of bound triplet pairs, we observe separation into charges with no sign of intermediate free triplets. Intriguingly, we find that the ^1^(TT) lifetime in neat and blend films is strikingly similar at 50 K. We can only reconcile this by citing the same rate-determining process for both ^1^(TT) separation to free triplets and charge separation from ^1^(TT): exciton diffusion to the interface. In blend films this is to a PC_71_BM-rich phase; in neat films diffusion is to grain boundaries or other defect sites (see above).

To determine qualitatively the contribution of ^1^(TT) and free triplets to the charge yield, we focus on Fig. 8f. Although the yield of free triplets varies significantly from 50 to 300 K, the charge yield remains constant. If charges were only generated from free triplets, their yield should track the free triplet yield. As this is not the case, we find that multi-electron transfer from ^1^(TT) should be the dominant pathway at low temperature.

## Discussion

We have demonstrated the general presence of the bound, entangled triplet-pair state ^1^(TT). An interesting property of ^1^(TT) is that it emits as a singlet but can transfer charge from each of its constituent triplets. We find that non-radiative decay of ^1^(TT) is a dominant loss mechanism limiting further exploitation. Similarly fast non-radiative ^1^(TT) decay has been observed in covalent acene dimers^24^,^63^, carotenoid aggregates^62^,^64^ and polymers^56^,^65^,^66^, and understanding the mechanism of this process and minimizing it could allow for efficient solar cells with direct charge transfer from ^1^(TT). Our work also has critical implications for TTA upconversion efficiency models^67^, where the implicit assumption is that the singlet-character TTA encounter complex (that is, ^1^(TT)) converts to S_1_ with unity efficiency. Instead we find that ^1^(TT) →S_1_ conversion efficiency is determined by the competition between singlet formation, triplet-pair separation and non-radiative decay.

More broadly, we consider that the generality of our findings across singlet fission materials affords a powerful platform to study the interactions between triplet excitons and the properties of bound multiexciton states and could lead to a new material set for solid-state quantum computing applications.

## Methods

### Materials

F_2_-TES ADT was synthesized as described previously^68^. PC_71_BM, tetracene, rubrene, TIPS-pentacene and pentacene were purchased from Sigma Aldrich. F_2_-TES ADT and TIPS-pentacene thin film samples were spin-cast (15 mg ml^−1^, toluene) on polyimide precoated fused-silica substrates (the polyimide aids wetting of the organic semiconductor). For F_2_-TES ADT: PC_71_BM thin film samples, 4:1F_2_-TES ADT:PC_71_BM blend solutions (15 mg ml^−1^ total material, mesitylene) were spin-cast on polyimide precoated fused-silica substrates. Samples were dried on a hot-plate at 50 °C for 10 min. Tetracene, rubrene and pentacene thin-film samples were prepared via thermal evaporation at a base pressure of <6 × 10^−6^ mbar.

Single crystals of F_2_-ADT were grown through a physical vapour growth method. Details of the relevant method can be seen elsewhere^69^. We used a growth apparatus equipped with source and growth heaters that were placed side-by-side. Temperatures of the heaters were regulated in such a way that high-quality single crystals could be produced. In the present studies we set the source and growth heaters at 240 °C and 220 °C, respectively. The growth duration was 6 h.

### TA and PL

For TA measurements, 90 fs pulses generated in a Ti:sapphire amplifier system (Spectra-Physics Solstice) operating at 1 kHz were used. The broadband probe beams were generated in separate home-built non-collinear optical parametric amplifiers for visible (500–800 nm) and near-infrared (800–1,100 nm) ranges. For fs-ps TA measurements, we used either narrowband <200 fs pulses from a commercial travelling-wave optical parametric amplifier of superfluorescence (TOPAS) or compressed pulses (∼40 fs, spanning 525–625 nm) from a home-built non-collinear optical parametric amplifier to excite the samples. The time delay between pump and probe pulses was controlled by a mechanical delay stage. Unless otherwise noted, pump intensities were kept below 5 μJ cm^−2^ to avoid bimolecular singlet–singlet annihilation effects and the pump and probe polarization were set to magic angle (54.7°) to avoid reorientation effects. For ns-TA measurements, pump pulses were generated using a frequency-doubled *Q*-switched ∼500 ps Nd:YVO_4_ laser (532 nm). Delay times from 1 ns to 1 ms were achieved using an electronic delay generator. For time-resolved PL measurements to generate spectral maps, the samples were excited by 40 ps pulses (excitation wavelength: 470 nm) operating at a repetition rate of 2.5 MHz. PL decay dynamics were resolved using electronic gating through time-correlated single-photon counting with a temporal resolution of 180 ps and detection based on a cooled microchannel plate photomultiplier tube coupled to a monochromator. PL quantum beating measurements were performed on a separate time-correlated single photon counting system (Becker-Hickl module) system, using 100 fs pulses at 500 nm with a repetition rate of 80 MHz. Here, the emission was detected with a silicon single-photon avalanche diode, yielding a temporal resolution of around 40 ps. For all solid-state time-resolved optical measurements, samples were kept at a fixed temperature in a cryostat with dynamic helium gas flow. For solution measurements, a fused silica 1 mm path length cuvette was used.

### Triplet sensitization

Blends of *N*-methylfulleropyrolidine (NMFP) and F_2_-TES ADT in a molar ratio of 4:1 were prepared at a concentration of 1 mg ml^−1^ in chloroform. The NMFP was excited by the 355 nm frequency tripled output of a *Q*-switched sub-ns Nd:YVO_4_ laser. Following intersystem crossing on NMFP, triplet transfer to F_2_-TES ADT occurs and is monitored by TA spectroscopy. Results are shown in Supplementary Fig. 11.

### Phosphorescence

To obtain samples that gave phosphorescence, the molecule of interest was spin-coated into films doped with platinum octaethylporphyrin (purchased from Sigma Aldrich). The weight ratio of target molecules to platinum octaethylporphyrin in solution was varied from 95:5 to 98:2, to give the clearest phosphorescence signal. Mixtures were spin-coated on Spectrosil at 800–1,200 r.p.m. for 1–2 min in a nitrogen-filled glove box and the films were then annealed at 60 °C for 30 min. The final samples were encapsulated to prevent sample degradation and triplet quenching by oxygen. Phosphorescence was detected using a calibrated infrared InGaAs photodiode array (ANDOR iDus 490A) coupled to a spectrograph (ANDOR Shamrock), with CW excitation at 532 nm (∼0.5 mW).

### Optically detected magnetic resonance

Optically detected magnetic resonance (ODMR) experiments were performed to investigate the presence of free triplets in F_2_-TES ADT. In these measurements, the change in PL is monitored under magnetic resonance. ODMR gives a direct way of identifying triplet excitons, as the dipole–dipole interaction between electron and hole spins within the triplet exciton gives rise to characteristically broad ODMR spectra, governed by the zero-field splitting Hamiltonian 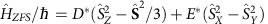
>, where 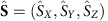 is the triplet spin operator, and D* and E* are the zero-field splitting parameters^70^.

Films were placed on a microwave stripline operated at a frequency of 6.2 GHz and mounted inside an optically accessible cryostat magnet, providing the static magnetic field *B*. Excitation was provided by a 532 nm laser with variable intensity *I*. The integrated PL was collected by a photodetector, after removing the laser line with a 550 nm long-pass filter. Microwaves were square-wave modulated at frequency *f*_*M*_, and the change in PL due to microwave transitions ΔPL was recorded by monitoring the photodetector response at this microwave chopping frequency using lock-in detection.

### Structural characterization

Specular X-ray diffraction was performed with a PANalytical Empyrean system in Bragg–Brentano geometry using a sealed copper tube (*λ*=1.5418 Å, 40 kV, 40 mA) and an X’Celerate detector operating in a one-dimensional mode. A variable slit optics choosing an illuminated length of 10 mm was used in combination with 0.04° Soller slits at the primary as well as at the secondary side. Low temperatures down to 85 K were reached with the low temperature attachment TTK600 from Anton Paar Ltd using vacuum (2 × 10^−5^ bar) conditions. Starting from room temperature the samples were cooled down in steps of 25 K with a cooling rate of 5 K min^−1^. After reaching a temperature of 85 K, the sample was rapidly heated up to room temperature and cooled down again in steps to a temperature of 230 K, to confirm the measurements were reproducible after thermal cycling. Careful alignment of the sample height and sample tilt were made to obtain reliable results. Peak parameters were determined by a fit of a Gaussian curve to the experimentally observed Bragg peaks.

For measurements conducted at room temperature, the out-of-plane film structure was investigated with X-ray reflectivity in a lab setup (D8, Bruker) using a wavelength of 1.5406 Å.

### Photovoltaic device fabrication and characterization

Solar cells were fabricated on 10 mm × 15 mm indium tin oxide-coated glass substrates that served as the anode. The substrates were ultrasonically cleaned in detergent, deionized water, acetone and isopropanol. A layer of 30 nm PEDOT:PSS (poly(3,4-ethylenedioxythiophene):poly(styrene sulphonate) (Clevios PH 1000) was spin-coated onto the indium tin oxide substrate and dried in air at 120 °C for 10 min. Fifteen milligrams of 4:1 F_2_-TES ADT/PC71BM blend was dissolved in 1 ml of mesitylene and spin-coated on top of the PEDOT layer at 1,500 r.p.m. and annealed for 10 min at 80 °C. Finally, the LiF/Al cathode (50 nm) was vacuum-evaporated onto the annealed photoactive layer. All devices were encapsulated before testing.

A 100 W tungsten halogen lamp (500–1,500 nm) and a 120 W Xenon lamp (350–500 nm) dispersed through a monochromator (Oriel Cornerstone 260) were used for external quantum efficiency measurements. For wavelengths between 375 and 900 nm, a set of silicon diodes (ThorLabs SM05PD1A) were used. A Keithley 2635 source measure unit was used to measure the short-circuit current as a function of wavelength. The incident light was focused to a spot size of ca. 1 mm^2^ using a set of lenses to illuminate individual pixels of size 0.08 cm^2^. The current–voltage (*I*–*V*) characteristics of the devices were measured under standard AM 1.5G conditions using an Abet Sun 2000 solar simulator, at an intensity equivalent to 100 mW cm^−2^. Spectral mismatch correction was performed before the measurements. The dark and bright *I*–*V* characteristics were measured using the Keithley 2635 source measure unit.

Internal quantum efficiency plots were calculated from external quantum efficiency (*λ*)/*A*(*λ*), where *A* is the absorbance of the photoactive layer, which was computed with a transfer matrix approach, where *A* was modelled according to published literature procedures^71^. The required values for the refractive index *n* and the extinction coefficient *k* were determined via ellipsometry, see Supplementary Fig. 25. The real and imaginary part of the complex refractive index were determined using variable angle spectroscopic ellipsometry (M-2000, Woollam Co.) using an optically isotropic layered optical model. The thickness was determined by fitting the data in the transparent wavelength range using the Cauchy model to describe the real part of the complex refractive index, assuming the imaginary part is zero. Film thicknesses were further determined via AFM.

### Spectral decomposition techniques

To identify the spectral species present in the TA measurements and to determine their individual evolution over time, we used a combination of singular value decomposition and a spectral deconvolution code^45^. This code generates a given number of spectra that best reproduce the original data, while satisfying basic physical constraints such as spectral shape and population dynamics. The optimization of these spectra is done by a genetic algorithm. Once the code has minimized the residual between the obtained spectra and the original data the output is a set of spectra and kinetics for the species identified. The advantage of this optimization method over other approaches is that the genetic algorithm starts from random initial spectra and does not require a starting kinetic scheme, such as in global analysis methods. In brief, the genetic algorithm is an example of an evolutionary algorithm that factors TA spectra into a pre-determined number of spectral components. The idea is based on natural selection of genes in a population; random spectra or parents are mixed until they give separate spectra that best reproduce the original data, which constitutes a test for genetic fitness. The advantages of a genetic algorithm for our purpose over other optimization methods, such as gradient search methods, is that it is more capable of modelling a multidimensional data space that can be noisy and contain several overlapping features. Gradient search methods, on the other hand, are much more likely to get stuck in local minima.

Here, this optimization code was used to analyse the separate spectral components of TA and PL spectra. Singular value decomposition was used to give an initial estimation of the number of principal components in each spectrum. From here the optimization code was run to look for the same number of species. We assumed that the initially photogenerated species primarily contains only singlets that evolve into other excited states at later time. Once it was determined whether two or three species best fit the data, the genetic algorithm was run several times on the same TA measurement to assess the reproducibility of the results. Unless otherwise mentioned, for fs–ps TA measurements, the algorithm was run over 200 fs to 2 ns. The precise dynamics of the sub-200 fs region are hard to assess due to the numerous fluctuations in the early-time signal and a time resolution of ∼200 fs. For ns-TA measurements, the algorithm was run over 3–2,000 ns, omitting the instrument response region. For ps-PL measurements, the algorithm was run over 200 ps–20 ns.

We assess the reliability of the results of the optimization in three ways: (1) by testing the reproducibility of the results from >10 runs of the genetic algorithm data, as shown in Supplementary Fig. 26; (2) by comparing the spectra obtained from the optimization to the raw data obtained from both TA and PL measurements; and (3) by comparing the kinetics of population decay and/or spectra with independent measurements, for example, comparing the singlet and ^1^(TT) intermediate state kinetics in the TA measurements with that in the transient PL measurements. These two measurements are both conducted in the linear regime at similar excitation fluence to ensure the same photophysics are observed in both measurements.

### *Ab initio* calculations

PL from the triplet pair state: to clarify the origin of ^1^(TT) PL, we analyse the bright exciton component in the ^1^(TT) state based on *ab initio* excited-state calculations. The ^1^(TT) states of *π*-stacked dimers are calculated using the complete active space self-consistent field (CASSCF) method, where the active orbitals consist of the highest-occupied molecular-orbital and lowest-unoccupied molecular-orbital of two monomers, that is CASSCF(4,4). The doubly excited ^1^(TT) diabatic state is optically dark and thus the ^1^(TT) PL necessitates mixing with singly excited bright exciton states. Yet, as found for the acene crystals^16^, the bright exciton component to the adiabatic ^1^(TT) states completely vanishes due to the relative phase factor of the wavefunctions in H-aggregates. In this case, the symmetric ^1^(TT) mixes only with the dark exciton and charge transfer states, which do not contribute to PL. However, the bright exciton can be mixed with ^1^(TT) via symmetry-breaking effects due to intra-molecular vibronic coupling. We model this in the simplest way possible by displacing the geometry of one of monomers from the triplet minimum to the ground state minimum, (see Fig. 2f in the main text for results and discussion). Such a scenario is consistent with intensity borrowing induced by coupling to vibrational modes, that is, the Herzberg–Teller mechanism.

Triplet pair separation rate: we analysed the transfer rate from the bound ^1^(TT) to free triplets based on the Marcus–Levich–Jortner rate theory parameterized against *ab initio* calculations. The electronic coupling for the intermolecular triplet transfer, *V*_T−T_, (see Fig. 5c in the main text) was estimated using time-dependent density functional theory as applied to *π*-stacked dimer models. The intramolecular reorganization energy, *λ*_intra_, for the triplet diffusion was computed on the monomers at the DFT level. The enthalpy change, Δ*H*, for the ^1^(TT) separation was obtained from CASSCF(4,4)/ CASPT2 calculations on *π*-stacked trimer models, with the monomers adopting either the fully relaxed triplet geometry (*T*) or the ground-state geometry (*G*). Thus, in this notation, the bound and separated triplet pairs correspond to TTG and TGT configurations, respectively.

The ^1^(TT) separation rate, *K*_T−T_, is evaluated by the Marcus–Levich–Jortner theory:


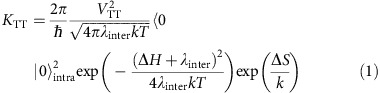


where *k*, *T* and *S* are the Boltzmann constant, temperature and entropy change, respectively. The Franck–Condon factor for the intramolecular effective mode, 〈0|0〉_intra_, is evaluated from the reorganization energy, *λ*_intra_, and the frequency of 0.18 eV corresponding to the usual breathing/stretching mode in aromatic/conjugated molecules. The low-frequency intermolecular reorganization energy, *λ*_inter_, is small for covalent excitations such as triplets and its calculation cumbersome. Here we take a conservative value of 0.05 eV; modifying it in a reasonable range (up to a factor of 3) hardly affects the conclusions below. eW account for Δ*S* considering the numbers of spatial and spin configurations for the bound ^1^(TT) and separated triplets. The ^1^(TT) separation pathway for *π*-stacked TIPS-pentacene, F_2_-TES ADT and rubrene can be regarded as a one-dimensional chain; in this case, the spatial Δ*S* from the bound ^1^(TT) to nearest-neighbour T+T is unity. Considering the dissipation of spin correlation, the number of spin configurations of the separated T–T (3 × 3) is three times as many as the total-singlet ^1^(TT), that is exp(Δ*S*/*k*)=3.

The calculated Δ*H* indicates that the ^1^(TT) separation is endergonic for the three molecular materials investigated (see Fig. 5d in the main text). The bound ^1^(TT) is stabilized by orbital delocalization as well as the admixture of singly excited electronic configurations into the nearest-neighbour TT pairs (TTG), both contributions vanishing in the case of separated triplets (TGT). The free-energy stabilization going from TGT to TTG completely dominates the activation energy Δ*E*. Although these are overestimated by the calculations, the predicted trends in the Δ*E* values (namely larger in TIPS-pentacene compared to rubrene) match reasonably well with the measurements. It is interesting to point out that for rubrene in its equilibrium crystal structure at 0 K, the singlet-triplet mixing is strictly null because of symmetry^20^; thus, the finite (yet smaller) Δ*E* value computed in that case reflects only the energy stabilization associated with direct wavefunction overlap between neighbouring triplets. The relatively large ^1^(TT) separation rate of TIPS-pentacene compared with F_2_-TES ADT and rubrene is rationalized by the difference in electronic coupling for triplet diffusion. Thus, based on these theoretical results, we believe the measured activation energies reported in Fig. 5 essentially reflect the energy stabilization of bound vs free triplet pairs, rather than the reorganization energy for the migration of free triplets. This is borne out by the fact that measurements in anthracene single crystals have demonstrated that the triplet diffusion coefficient is quasi temperature-independent in the range 100–300 K (ref. 72) (see also references therein).

### Data availability

The data that support the findings shown in both the main figures and Supplementary Figures in this study are available with the identifier https://doi.org/10.17863/CAM.9032.

## Additional information

**How to cite this article:** Yong, C. K. *et al*. The entangled triplet pair state in acene and heteroacene materials. *Nat. Commun.* **8**, 15953 doi: 10.1038/ncomms15953 (2017).

**Publisher’s note:** Springer Nature remains neutral with regard to jurisdictional claims in published maps and institutional affiliations.

## Supplementary Material

Supplementary Information

## Figures and Tables

**Table 1:** Diabatic mixing in ^1^(TT).

	**Bright S**_**1**_	**Dark S**_**1**_	**CT**
TIPS-Pn (T T_equilibrium_)	0.00	0.28	1.34
TIPS-Pn (T G_equilibrium_)	0.22	0.10	1.22
F_2_-TES ADT (T T_equilibrium_)	0.00	0.24	0.96
F_2_-TES ADT (T G_equilibrium_)	0.46	0.02	0.08

Mixing ratios (%) of other diabatic states into ^1^(TT) in two geometries.

^1^(TT) is optically dark at equilibrium (T T_equilibrium_). Distortion of one molecule (T G_equilibrium_) activates mixing with the bright singlet and thus ^1^(TT) emission, exemplifying Herzberg–Teller coupling. Details in the Methods section.

**Table 2:** Parameters for triplet hopping model from *ab initio* calculations.

	***V***_**T−T**_ **(meV)**	**Δ*****H*** **(meV)**	**Δ*****E*** **(meV) at** ***λ***_**inter**_**=50** **meV**	***λ***_**intra**_ **(meV)**
TIPS-Pentacene	22	87	94	177
F_2_-TES ADT	33	67	68	195
Rubrene	18	47	47	242

Electronic coupling for triplet transfer *V*_T−T_, enthalpy of ^1^(TT) separation Δ*H*, activation energy Δ*E* and intramolecular reorganization energy for triplet diffusion *λ*_intra_, in units of meV. Details in the Methods section.
