# Supplementary material for: The entangled triplet pair state in acene and heteroacene materials
Source: Nat Commun. 2017 Jul 12;8:15953. doi: 10.1038/ncomms15953 (PMC5510179; doi:10.1038/ncomms15953)
Supplement: Supplementary Information [file ncomms15953-s1.pdf]

File name: Supplementary Information
Description: Supplementary Figures, Table and Notes

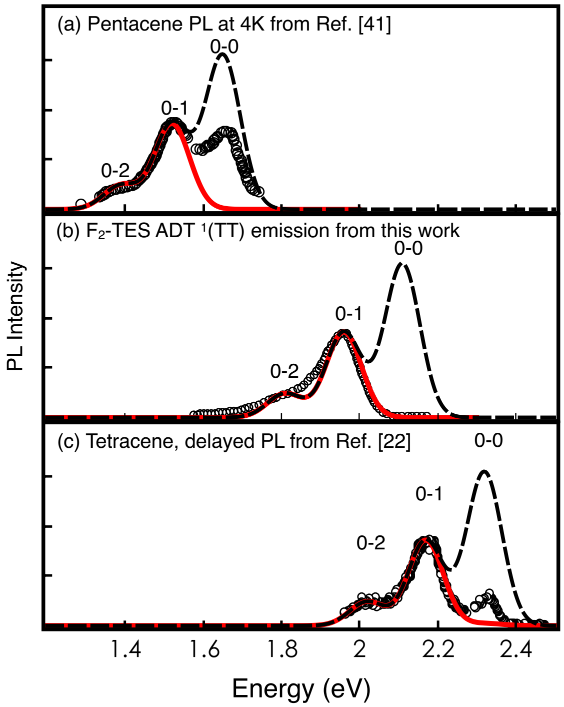

Supplementary Figure 1: **Triplet-Pair 0–0 Energy.** To estimate the energy of ^{1}(TT), we use fits to the PL spectra. The figure shows the PL spectrum (markers) of (a) a pentacene film from Ref. [53], (b) a delayed PL spectrum of a tetracene film from Ref. [74], and (c) the ^{1}(TT) emission spectrum of F_2-TES ADT from this work (fig. 2 in the main text). The lines are multi-Gaussian fits to the data. According to our model of ^{1}(TT) emission described in the main text (the Herzberg-Teller approximation), the ^{1}(TT) 0–0 emission is suppressed and the prominent feature in the ^{1}(TT) spectrum is the 0–1 vibronic replica. To estimate the 0–0 energy of ^{1}(TT), we fit the 0–1 and 0–2 features to a pair of Gaussians of identical width. The spacing between the peaks is then assumed to be the effective vibrational energy. We used Gaussian functions ($y = A\sqrt{2/\pi}/w \exp(-2((x - x_c)/w)^2)$) with constant width ($w$ =0.09 eV) and even spacing between vibronic replica (0.13 eV in pentacene, 0.15 eV in F_2-TES ADT and tetracene). Using this method, we find ^{1}(TT) 0–0 energies of 1.65 eV (pentacene), 2.11 eV (F_2-TES ADT) and 2.32 eV (tetracene). It is interesting that some 0–0 emission is observed in both pentacene and tetracene, possibly due to stronger intensity borrowing from the singlet or increased disorder compared with F_2-TES ADT. The main source of error in the extracted ^{1}(TT) energy is the error in estimating the effective vibrational energy, estimated to be $\pm$0.01 eV. It is also striking that the 0–1/0–2 ratio is similar in all three materials, measured as I_{0-1}/I_{0-2}=3.6. This suggests similar emissive species in each with a Huang-Rhys parameter of S=0.56. We have plotted both fully suppressed 0–0 (red) and fully allowed 0–0 (black dashed) assuming a Huang-Rhys parameter of S=0.56.

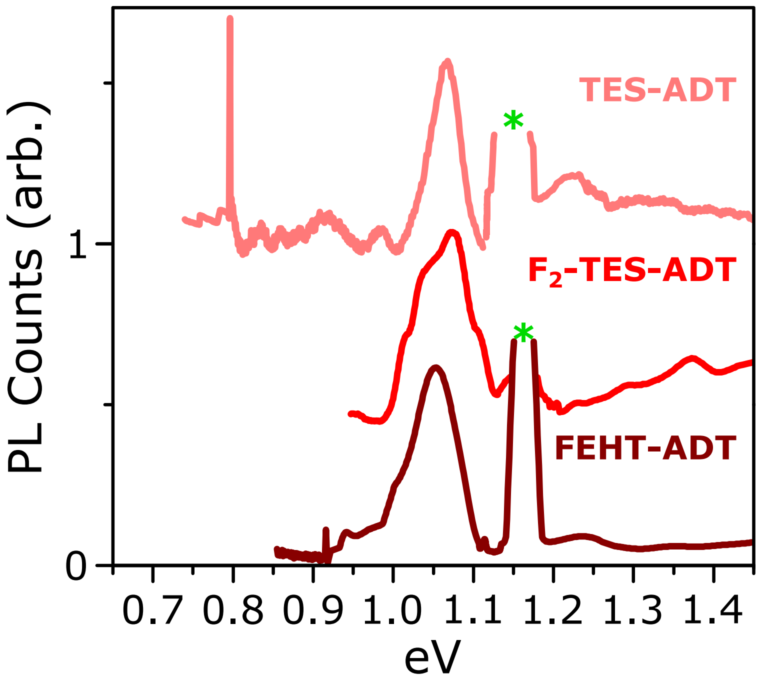

Supplementary Figure 2: **ADT film phosphorescence**. As described in the Methods, we measured the triplet energy in films using sensitisation with platinum octaethylporphyrin. Here we show phosphorescence of three anthradithiophene derivatives: TES-ADT, F_2-TES ADT and FEHT-ADT with excitation at 532 nm (2.33 eV). From this, the triplet energy of F_2-TES ADT is taken to be 1.08 eV. This energy is used in fig. 4 in the main text. It is interesting that the molecular structure of the anthradithiophene derivative doesn't greatly alter the triplet energy. The green star represents an artefact from the laser at 1.17 eV.

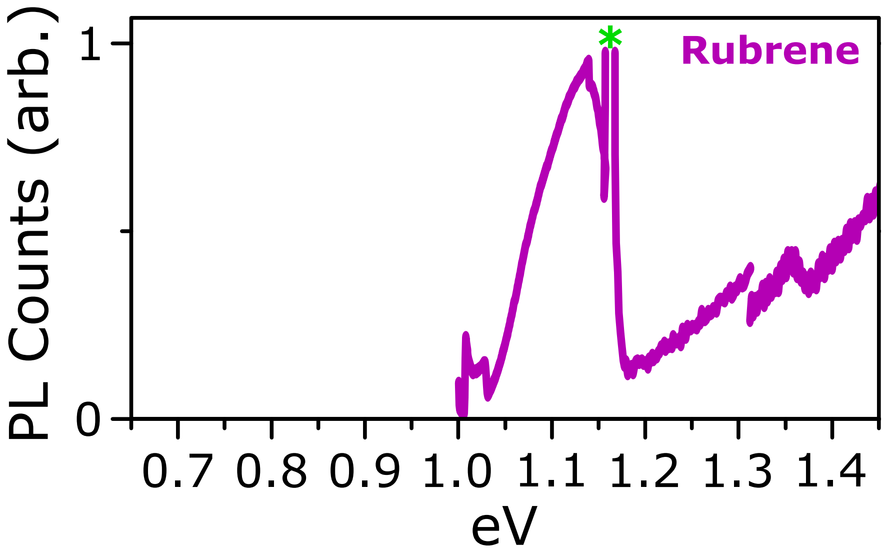

Supplementary Figure 3: **Phosphorescence of a rubrene film sensitised with platinum octaethylporphyrin** with excitation at 532 nm, see Methods Section in the main text. The triplet energy in rubrene is therefore taken to be 1.14 eV and is used in Fig. 4 of the main text. The green star represents an artefact from the laser at 1.17 eV.

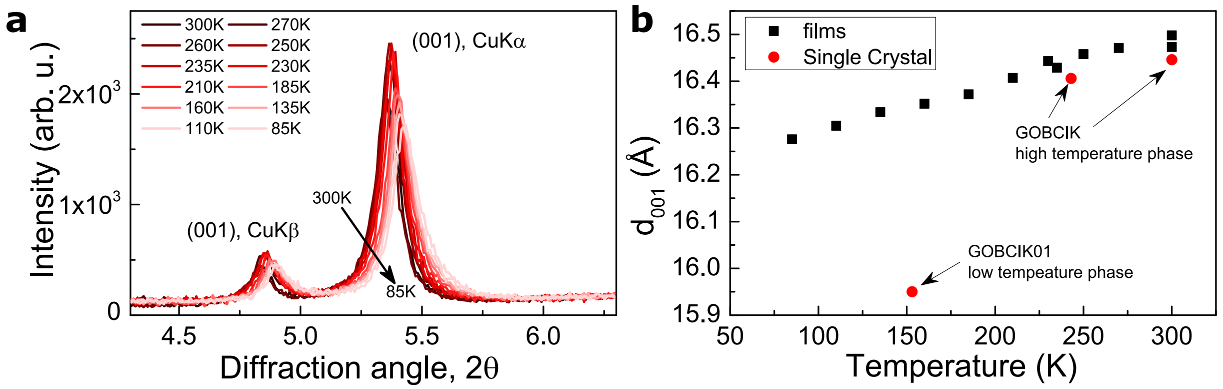

Supplementary Figure 4: **F_2-TES ADT film X-ray diffraction.** (a) X-ray diffraction of F_2-TES ADT films at different temperatures. (b) Lattice parameter of the (001) plane of F_2-TES ADT films (black squares) as a function of temperature. The lattice parameters of the (001) plane of F_2-TES ADT single crystals at different temperatures are shown as red circles. Specular X-ray diffraction at room temperature reveals a strong Bragg peak at a scattering angle of $2\theta = 5.356°$ ($d = 16.50\mathring{A}$) and higher order reflections of this peak up to the fourth order. The peak at $2\theta = 4.835°$ is due to the illumination of samples by Cu-Kβ. Since no further peaks are observed a strong preferred orientation of the crystallites can be concluded. This peak can be assigned to the (001) plane of the high temperature phase of the molecule [69]. Low-temperature investigations of the 001 Bragg peak reveal a peak shift to larger 2θ values (smaller d values). Supplementary Figure 4 shows the interplanar distance d_{001} as a function of temperature down to 85 K: a linear behaviour is observed. The results are in excellent agreement with calculated values of d_{001} based on the reported crystal structures at temperatures of 245 K and 295 K [69]. The d_{001} decreases gradually as the temperature is reduced. In marked contrast, a phase transition has been reported in single crystals with an abrupt change of d_{001} at temperatures below ~ 150 K[75]. The experimental results thus establish that, for spin-cast F_2-TES ADT films, the d_{001} gradually reduces as the temperature is decreased and, unlike single crystal, no significant phase transition appears in the thin film at low temperatures. This is consistent with our temperature-dependent absorption and PL spectra in Figure 1 of the main text which show a monotonic change, with no evidence of abrupt shifts.

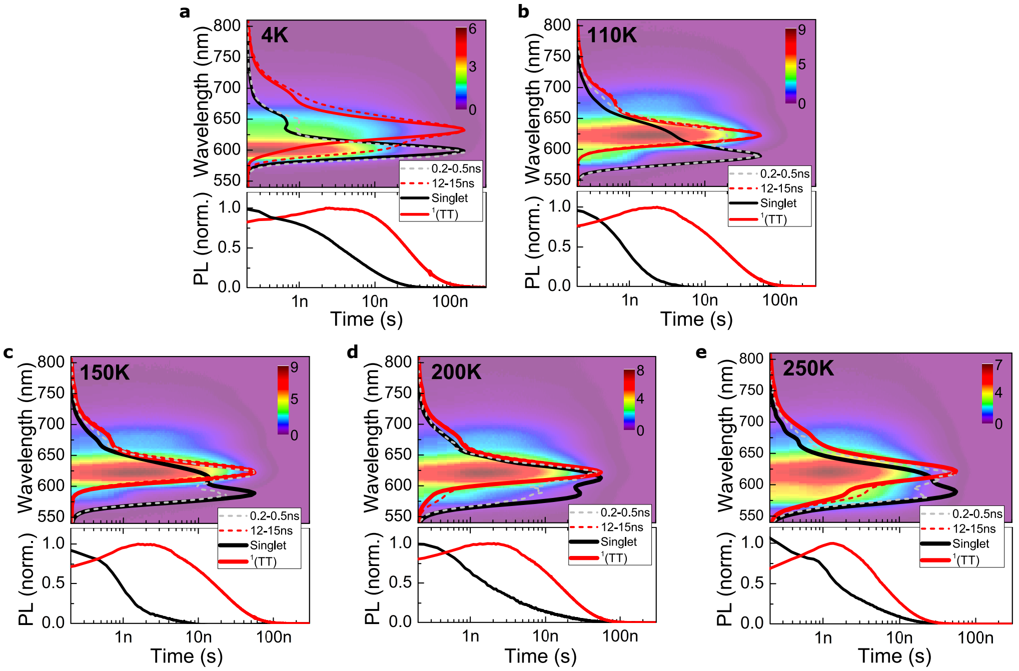

Supplementary Figure 5: **Time-resolved PL maps for F_2-TES ADT films** taken at 4 K–250 K (a–e), 50 K and 300 K are shown in the main text. As described in the main text, we observed the growth of ${}^1(\mathrm{TT})$ emission peaking at $\sim$630 nm, which occurs as the singlet decays. Spectral decomposition confirms the presence of two PL species - singlet (black curves) and ${}^1(\mathrm{TT})$ (red curves). The extracted spectra closely match the raw data (dashed lines). The bottom panel of each PL map shows the corresponding population kinetics (normalised).

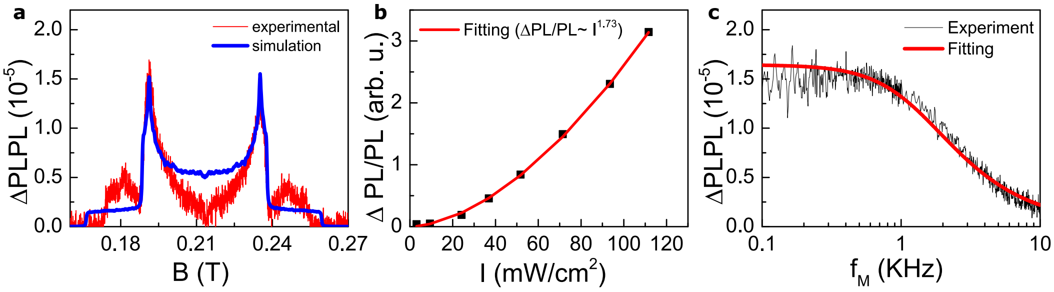

Supplementary Figure 6: **F_2-TES ADT film Optically Detected Magnetic Resonance (ODMR) spectroscopy** also discussed in Supplementary Note 2 (a) ODMR at 45 K along with a fit to a density matrix model, identifying triplet exciton with zero-field splitting parameters of $D^* = 1.30$ GHz and $E^* = 0.03$ GHz. (b) Laser intensity dependence of the ODMR signal showing bimolecular kinetics. (c) Microwave chopping frequency dependence of the ODMR signal along with a fit to an exponential relaxation model which yields a characteristic lifetime of $\tau = 118\,\mu$s..

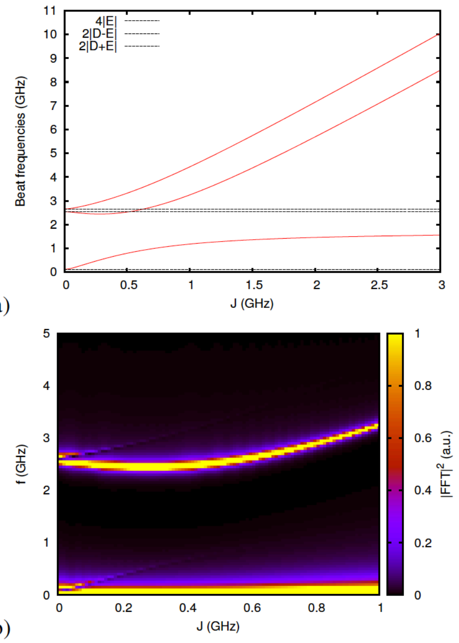

Supplementary Figure 7: **F_2-TES ADT calculated beat frequencies as a function of triplet interaction strength.** Quantum beats are described in the main text and in Supplementary Note 1: (a) Beat frequencies as a function of interaction strength, J (dotted lines: interaction-free). (b) Simulated Fourier spectra. Note that both frequencies and relative amplitudes shift with increasing J.

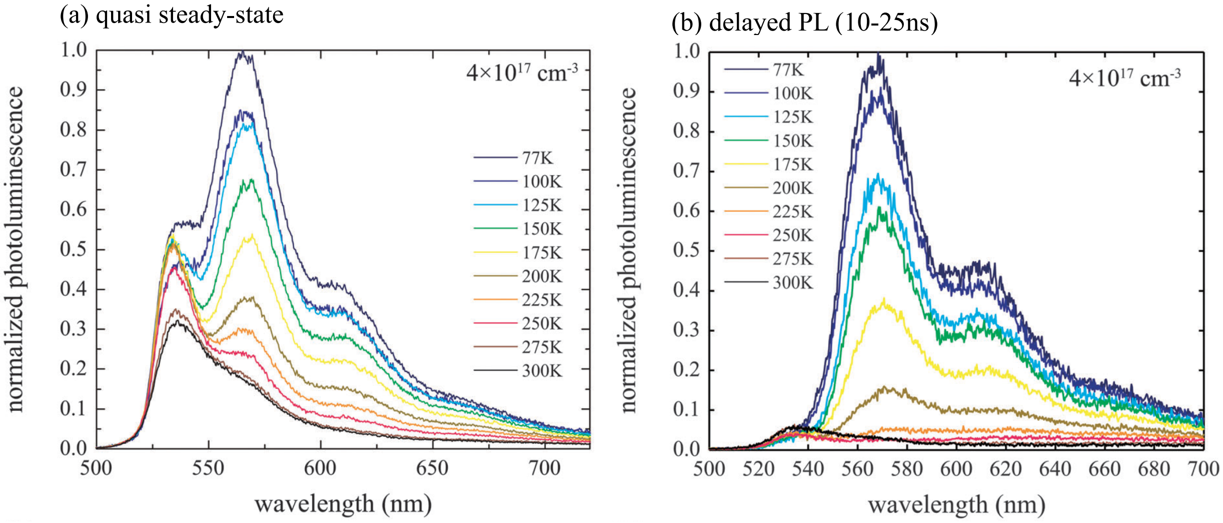

Supplementary Figure 8: **PL of tetracene polycrystalline films. Figures from** [34] **- Published by the PCCP Owner Societies, used with permission.** See discussion in Supplementary Note 3. (a) Quasi-steady state PL and (b) delayed PL spectra of tetracene thin films with exciton density of 4×10^{17} cm^3 and a pump wavelength of 500 nm. The data is normalised to the peak of the 77 K spectrum in both cases and highlights the relative increase of a feature at $\sim$570 nm as the film is cooled. This feature dominates the delayed PL spectra in (b) and we attribute it to ^{1}(TT).

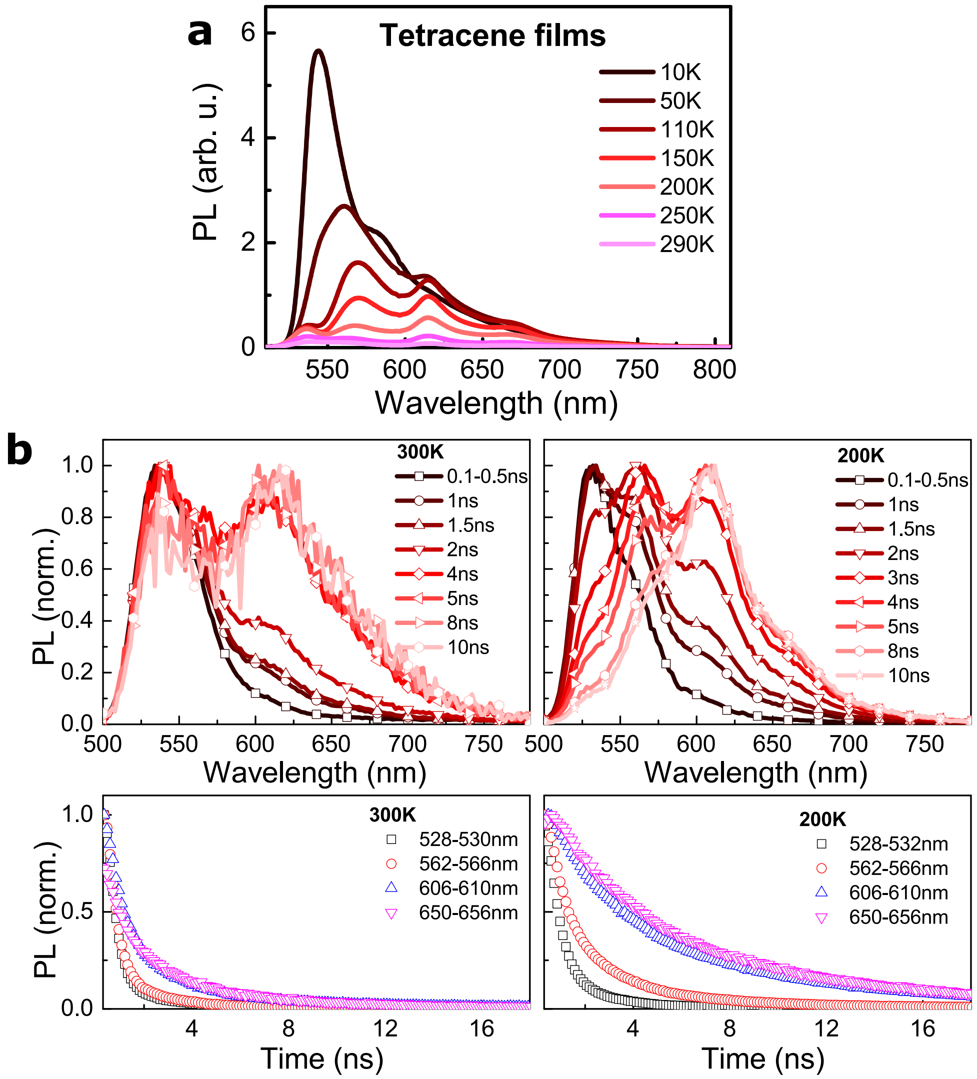

Supplementary Figure 9: **PL of tetracene polycrystalline films.** See discussion in Supplementary Note 3. (a) Steady-state temperature-dependent PL spectra. Similar to Supplementary Figure 8, as the film is cooled from 300–100 K, the 0–1 ^{1}(TT) vibronic replica at 570 nm grows relative to S_1. Cooling further leads to an enhancement of S_1 0–0. Compared with Supplementary Figure 8, the increased intensity between 600-700 nm is due to overlapping excimer emission [46]. (b) PL spectra at the time-delays marked in the figure at 300 K and 200 K, normalised to the maximum signal. Bottom panels: PL decay of tetracene thin films with 100 ps excitation at 530 nm (S_1 peak), 650 nm (very little S_1) and two intermediate wavelengths.

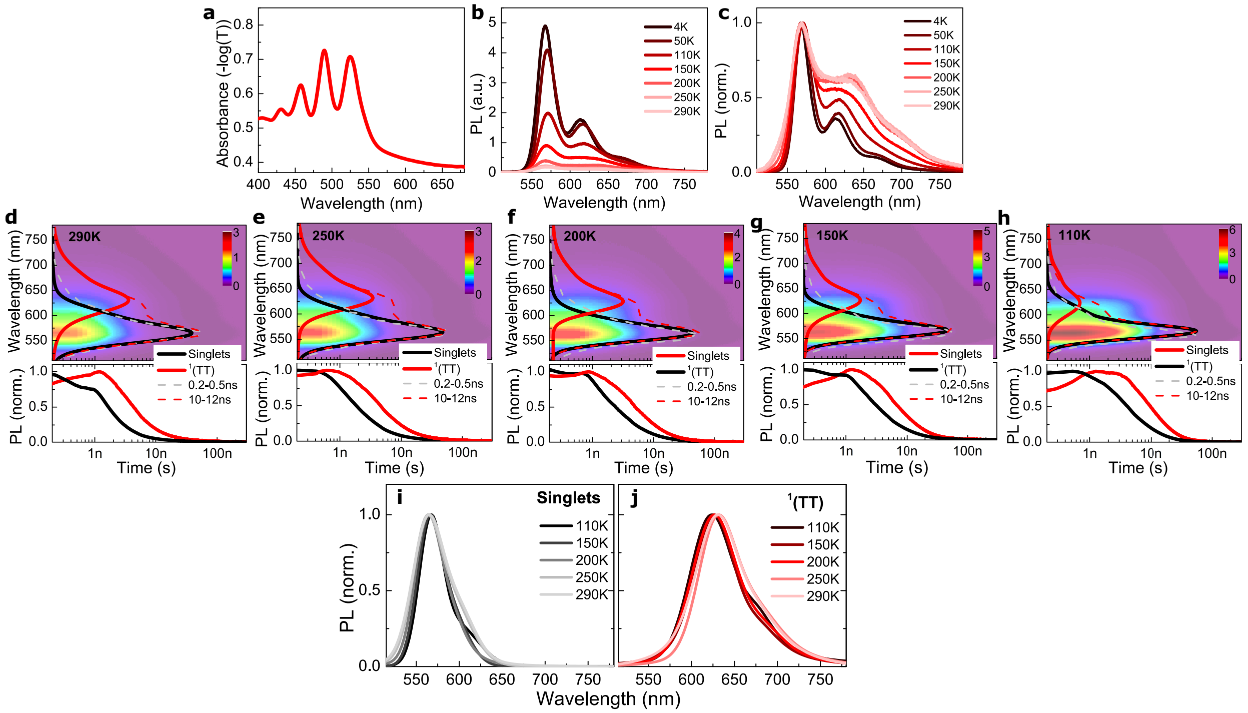

Supplementary Figure 10: **Rubrene film PL spectroscopy.** Singlet exciton fission has previously been reported in rubrene, occurring with a time constant of ∼ 100 ps[76]. Our samples were prepared via thermal evaporation with a base pressure maintained at 2×10^{-6} mbar creating ∼80 nm thick films. (a) shows the absorption spectrum of a rubrene film at room temperature and (b) shows the emission spectra of rubrene films from 4 K to room temperature (normalised in panel (c) to the 0–0 emission peak). Comparing the CW absorption and PL spectra in with previous work [39] suggests the film is predominantly amorphous. The spectra also show that, similar to F_2-TES ADT and tetracene [49], a temperature-dependent red-shifted feature is apparent peaking at ∼625 nm. The time-resolved PL maps taken at (d) 290 K, (e) 250 K and (f) 200 K, (g) 150 K, and (h) 110 K show the red-shifted feature to be long-lived. Based on the assignment of this red-shifted state in F_2-TES ADT, we perform spectral decomposition to extract two species, shown as solid lines. The corresponding population kinetics are shown in the lower panels of (d)-(h) and extracted emission spectra are shown in (i) and (j), respectively. The decomposition shows strong overlap between prompt and delayed emission profiles even at the lowest temperatures, making it difficult to distinguish the 'pure' singlet and ^{1}(TT) contributions from these kinetic components. From the agreement with TA kinetics in fig. 14, we are confident that the delayed PL dynamics are governed by the ^{1}(TT) state and we label it as such, but the spectral congestion here prevents a definitive determination of the true ^{1}(TT) PL spectrum.

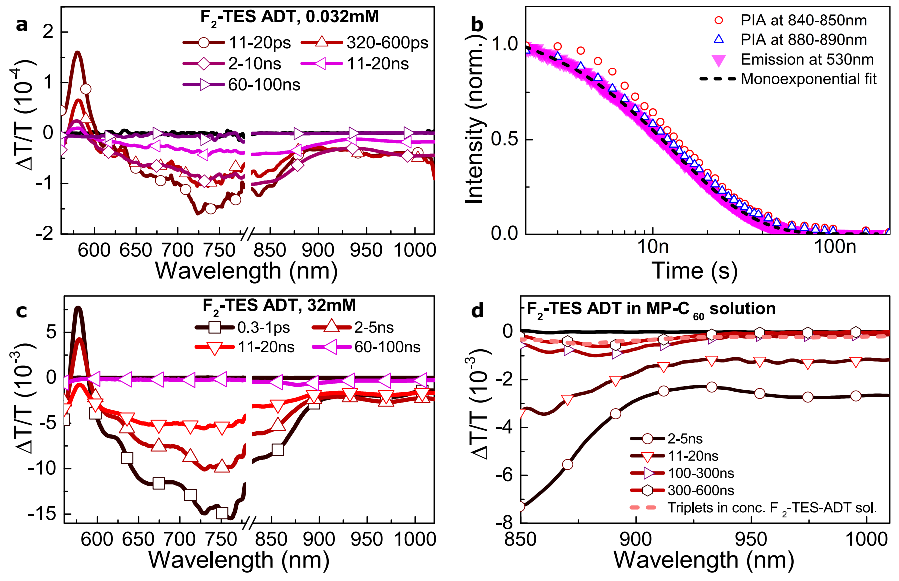

Supplementary Figure 11: **TA spectra of dilute and concentrated solution samples of F_2-TES ADT in chloroform.** (a) and (b) show dilute solution (0.032 mM) spectra and dynamics at selected wavelengths, compared with emission dynamics at 530 nm and monoexponential fit with $\tau = 12$ ns. In dilute solution, the photoexcitations decay with only minor spectral relaxation and with similar kinetics in both TA and emission, confirming the dominant photogenerated species as singlet excitons. (c) Concentrated solution (32 mM) spectra and (d) sensitisation studies on F_2-TES ADT:NMFP blend solution. Final spectrum (F_2-TES ADT triplet, red line) shows good spectral overlap with final spectrum in (c) (dashed red line) demonstrating presence of triplet excitons from singlet fission in concentrated F_2-TES ADT solution. We assign this to formation of triplets from singlet exciton fission, see main text. As suggested previously, the kinetics of singlet fission in solution depend mainly on the diffusion of molecules in the solution [35].

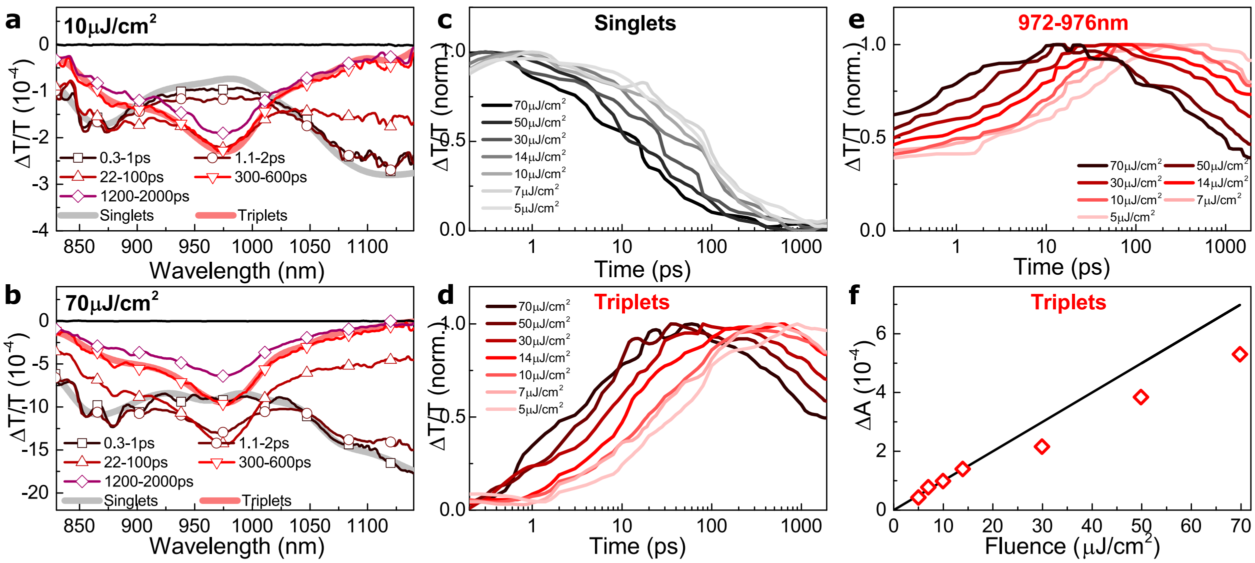

Supplementary Figure 12: **Excitation-density dependent effects on the transient absorption kinetics of F_2-TES ADT films.** We excited the sample at 530 nm at 300 K with a range of excitation fluences and performed spectral decomposition to extract singlet and triplet dynamics. Parts (a) and (b) show the TA spectra of F_2-TES ADT films taken at an excitation fluence of (a) 10 μJcm^{-2} and (b) 70 μJcm^{-2}. (c) and (d) show the population kinetics of singlets and triplets, respectively, extracted from spectral decomposition analysis. Panel (e) shows raw data kinetics extracted at wavelengths near the peak of the triplet absorption spectrum (972–976 nm). From spectral decomposition, the maximal ΔA of triplets at different excitation fluences is shown in (f). Above 20 μJcm^{-2}, ΔA grows sub-linearly with the excitation fluence, indicating the presence of non-negligible singlet-singlet annihilation that competes with singlet exciton fission. To ensure our experiments were carried out under the linear regime in the absence of singlet-singlet annihilation, we excited our samples at excitation fluences below 5-10 μJcm^{-2}. As demonstrated below and in the main text, under such excitation conditions, the kinetics of singlet fission measured with TA closely match with those observed in PL, where samples were subjected to a 40 ps excitation pulse with a maximal excitation fluence of 3 μJcm^{-2}.

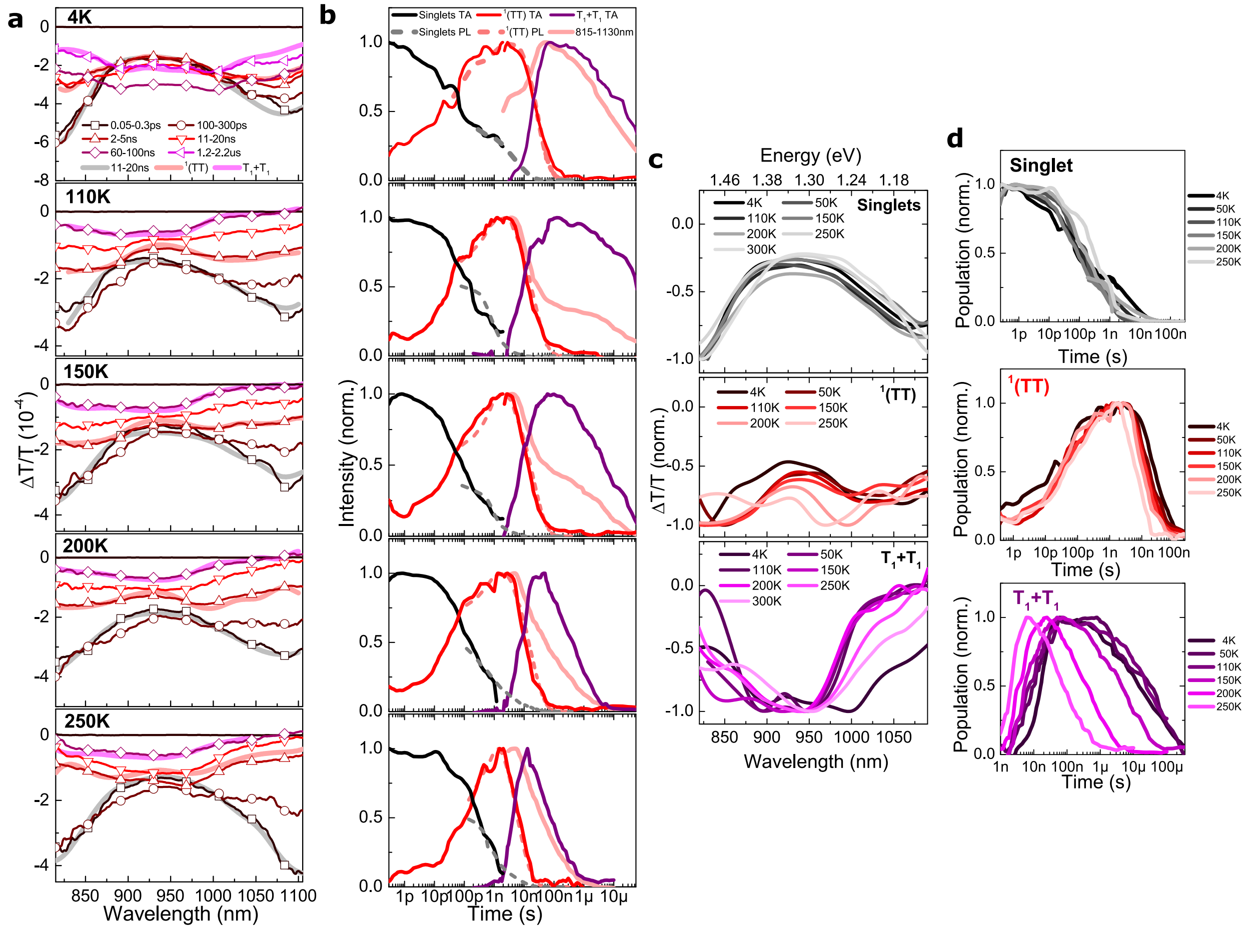

Supplementary Figure 13: **TA of F_2-TES ADT films.** (a) and (b) show the TA spectra and dynamics measured at 4 K, 110 K, 150 K, 200 K and 250 K (300 K and 50 K are found in the main text). Spectral decomposition analysis shows the presence of three kinetically distinct species - shown by thick black, red and purple lines in (a) and (b). The raw kinetic traces integrated over the entire NIR spectral range are overlayed with these in pink and the dashed lines show corresponding kinetics extracted from PL dynamics in Supplementary Fig. 2. As described in the main text, the initial TA spectra are assigned to singlets (thick black lines) and an intermediate emissive species is assigned to the spin-coherent triplet-pair ^{1}(TT) state (thick red lines). The extracted spectra and corresponding population dynamics are shown as a function of temperature in (c) and (d) respectively.

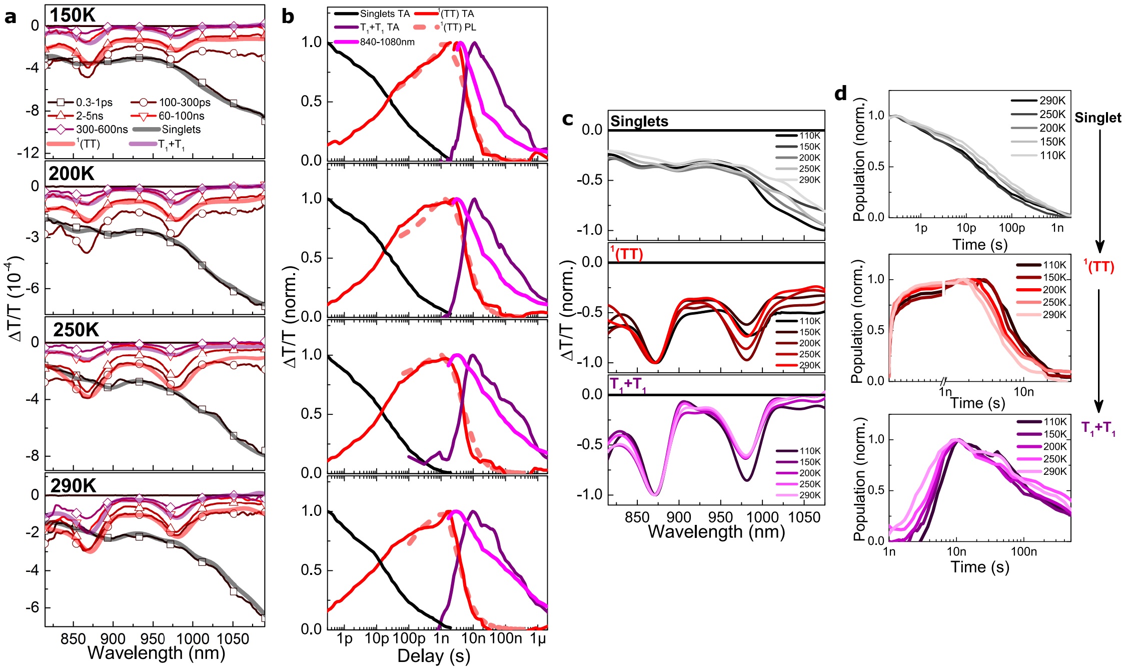

Supplementary Figure 14: **TA of rubrene films.** (a) TA spectra of rubrene films excited at 530 nm and measured at 150 K, 200 K, 250 K and 290 K. At all temperatures measured, the raw kinetic data integrated across the NIR spectrum (pink solid lines in Supplementary Figure 14b) shows a long-lived feature which is not emissive. Spectral decomposition once again separates the spectra into three distinct kinetic species. The striking similarity between this and F_2-TES ADT in Supplementary Figure 13 allows us to assign the initial state to emissive singlets (black thick lines), the intermediate to emissive $^1(\mathrm{TT})$ (red thick lines) and the final state to free triplets (purple thick lines). The kinetics of singlet and $^1(\mathrm{TT})$ populations extracted from the TA measurements are shown in (b). They closely follow the kinetics extracted from the independently measured PL maps, confirming the spectral decomposition methods are consistent. Figures (c) and (d) show the temperature-dependent extracted spectra and population kinetics. Further confirmation of the presence of an emissive $^1(\mathrm{TT})$ state is the presence of the expected large negative offset of the $^1(\mathrm{TT})$ photoinduced absorption spectrum which is thought to demonstrate combined singlet and triplet photoinduced absorption features. Such behaviour has previously been reported in TIPS-tetracene solutions [21].

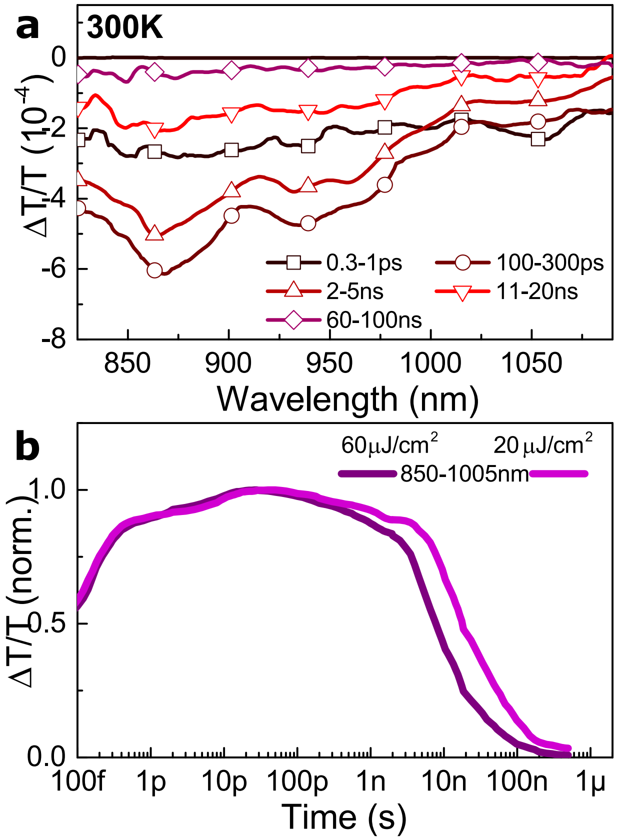

Supplementary Figure 15: **TA of TIPS-pentacene at 300 K.** Singlet exciton fission in TIPS-pentacene with a time-constant of ∼ 120 fs is well established [38]. The low free triplet pair energy with respect to the lowest-lying singlet exciton energy gives significant exothermicity for singlet fission. While the process has been widely studied with TA spectroscopy, there is no clear optical evidence of the bound triplet pair ^{1}(TT). To determine whether our model of bound ^{1}(TT) applies as well to exothermic systems, we conducted temperature-dependent TA measurements on TIPS-pentacene. The overall temporal resolution of the setup was ∼ 200 fs. (a) TA spectra of TIPS-pentacene, averaged over the indicated time periods. The initial sub-picosecond spectral evolution (within the instrument response time) is consistent with the typical ∼100 fs fission timescale [38]. At room temperature, the long-time photoinduced absorption spectra are consistent with free triplets [38]. (b) Decay kinetics averaged over the full triplet PIA band 850 nm-1005 nm at two different excitation densities. The acceleration of triplet decay at higher intensity is a sign of bimolecular triplet-triplet annihilation [77].

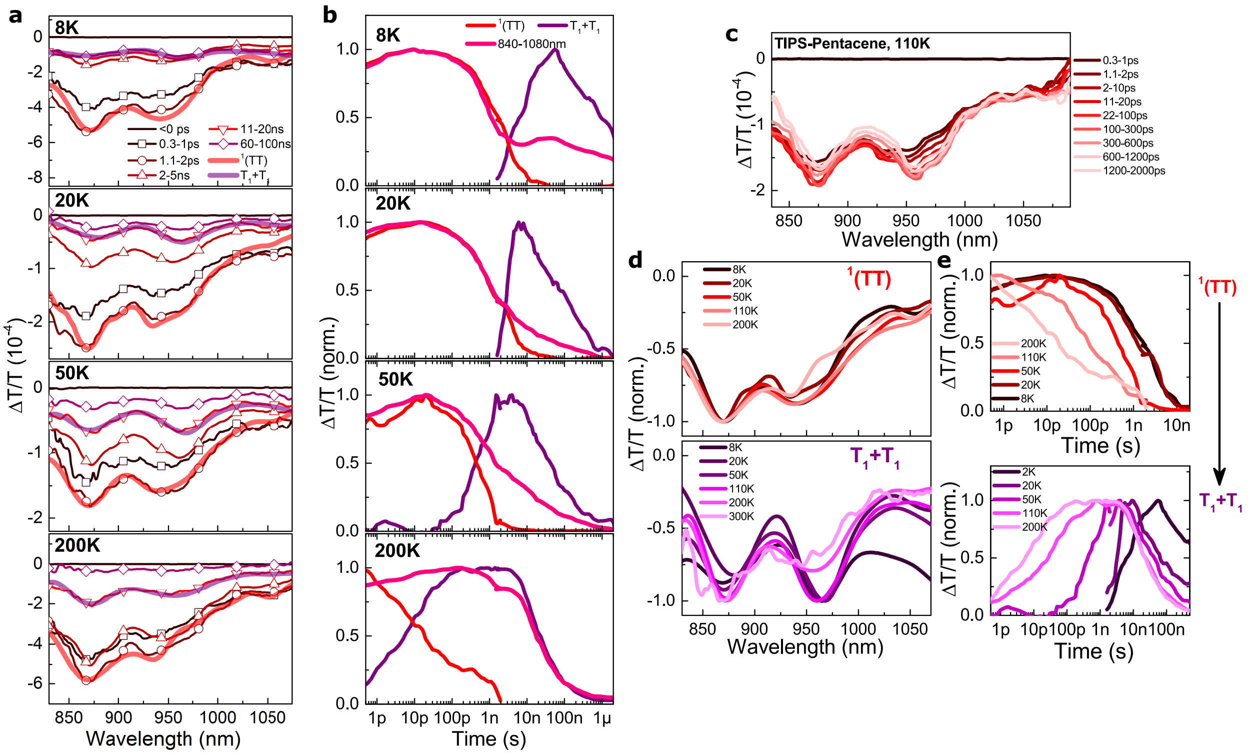

Supplementary Figure 16: **Low-temperature TA of TIPS-pentacene.** See also discussion in Supplementary Note 4. (a) TA spectra of TIPS-pentacene films measured at 8 K, 20 K, 50 K and 200 K. Since the time-constant of singlet fission is comparable to our instrument temporal resolution, we analysed the datasets only beyond 500 fs, where the singlet population is fully depleted and the system is dominated by triplets. For measurements conducted at temperatures below 200 K, spectral decomposition analysis reveals the presence of two kinetically distinct species, which, in comparison with F_2-TES ADT and rubrene, we assign to ^{1}(TT) (thick red lines) and free triplets (thick purple lines). The corresponding population kinetics and raw dynamics (pink) are shown in (b). Figure (c) shows raw spectra at 110 K highlighting the spectral evolution. The temperature-dependent extracted spectra and kinetics are shown in (d) and (e). We are unable to observe any ^{1}(TT) emission in this system. However, the transient absorption spectra of ^{1}(TT) resembles that observed in rubrene shown above, where the sharp triplet peaks are superimposed onto a broad background. These combined observations thus allow us to assign the absorption spectra summarised in the top panel of (d) to the ^{1}(TT) state. The absorption spectra of free triplets are characterised by sharp features very similar to those previously observed in concentrated TIPS-pentacene solutions [36]. The temperature dependence for the formation of free triplets is much more pronounced than in other materials and demonstrates that the model established for F_2-TES ADT also applies to this exothermic system. From the temperature dependence we can extrapolate a free triplet formation time constant of ∼200 fs at room temperature, which explains why it has not been clearly observed previously [22]. Very similar conclusions can be drawn from the results on evaporated pentacene films, presented below.

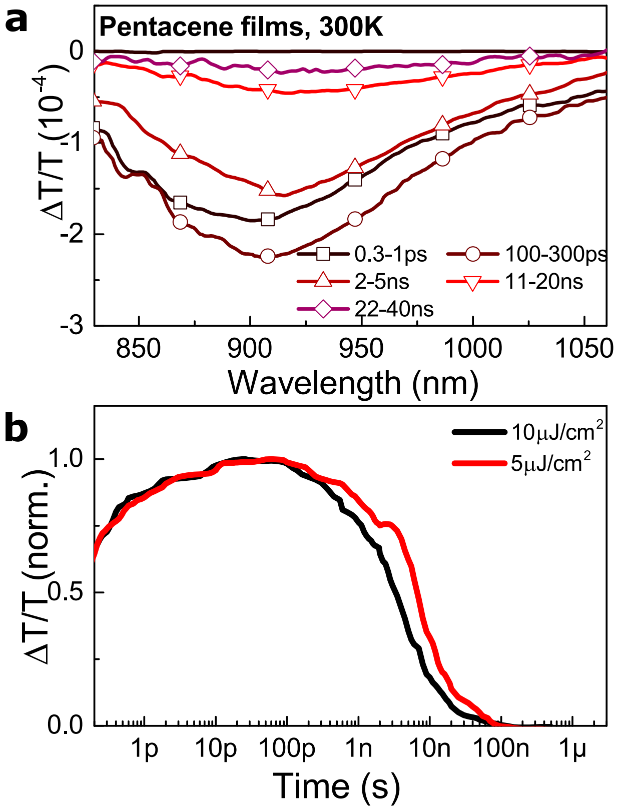

Supplementary Figure 17: **Pentacene film TA spectroscopy, 300 K**. Singlet exciton fission in pentacene with a time constant of ~ 80 fs is well established [38,61]. Similar to TIPS-pentacene, no clear demonstration of a $^1(\mathrm{TT})$ intermediate state has been revealed in ultrafast optical measurements [22], [78]. Here, we conducted temperature-dependent TA measurements on pentacene films prepared by thermal evaporation, again with a temporal resolution of ~ 200 fs. (a) TA spectra of pentacene films measured at 300 K. Very little spectral change was observed beyond 1 ps. The broad photoinduced absorption spectra observed here can be assigned to free triplets. (b) The kinetics of triplet recombination averaged over the wavelength range 900 nm–1000 nm at two excitation fluences. The triplet recombination is dominated by triplet-triplet annihilation[77].

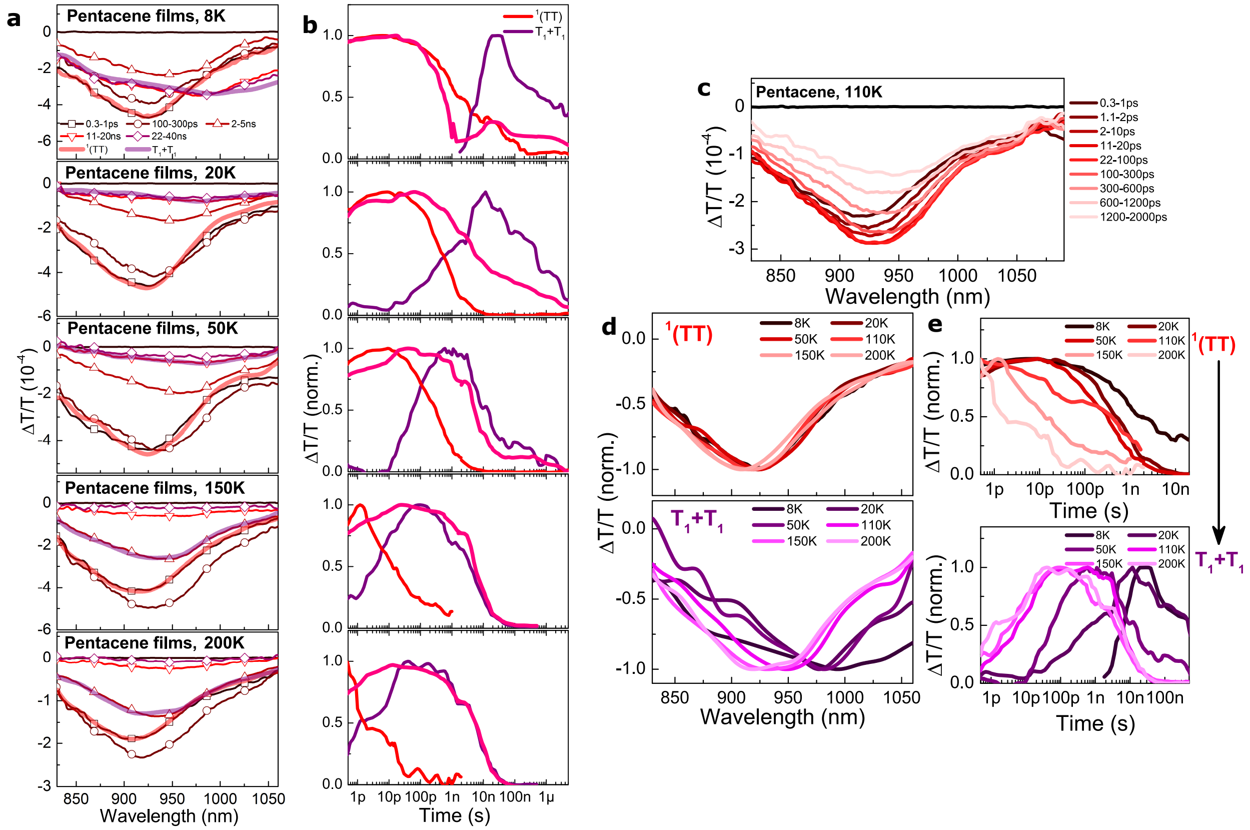

Supplementary Figure 18: **Low-temperature TA of pentacene films.** See also discussion in Supplementary Note 4 (a) TA spectra of pentacene films measured at 8 K, 20 K, 50 K, 150 K and 200 K. We analysed the data sets as for TIPS-pentacene above. Spectral decomposition analysis confirms the presence of two kinetically distinct species: ^{1}(TT) (thick red lines) and free triplets (thick purple lines). The kinetics of ^{1}(TT) and $T_1 + T_1$ extracted from the spectral decomposition analysis are shown in (b) along with raw data integrated across the NIR absorption band (pink). (c) Temporal evolution of the transient absorption spectra of pentacene measured at 110 K. Transient absorption spectra (d) and dynamics (e) of ^{1}(TT) and free triplets $T_1 + T_1$ in pentacene films extracted from the spectral decomposition methods as described above.

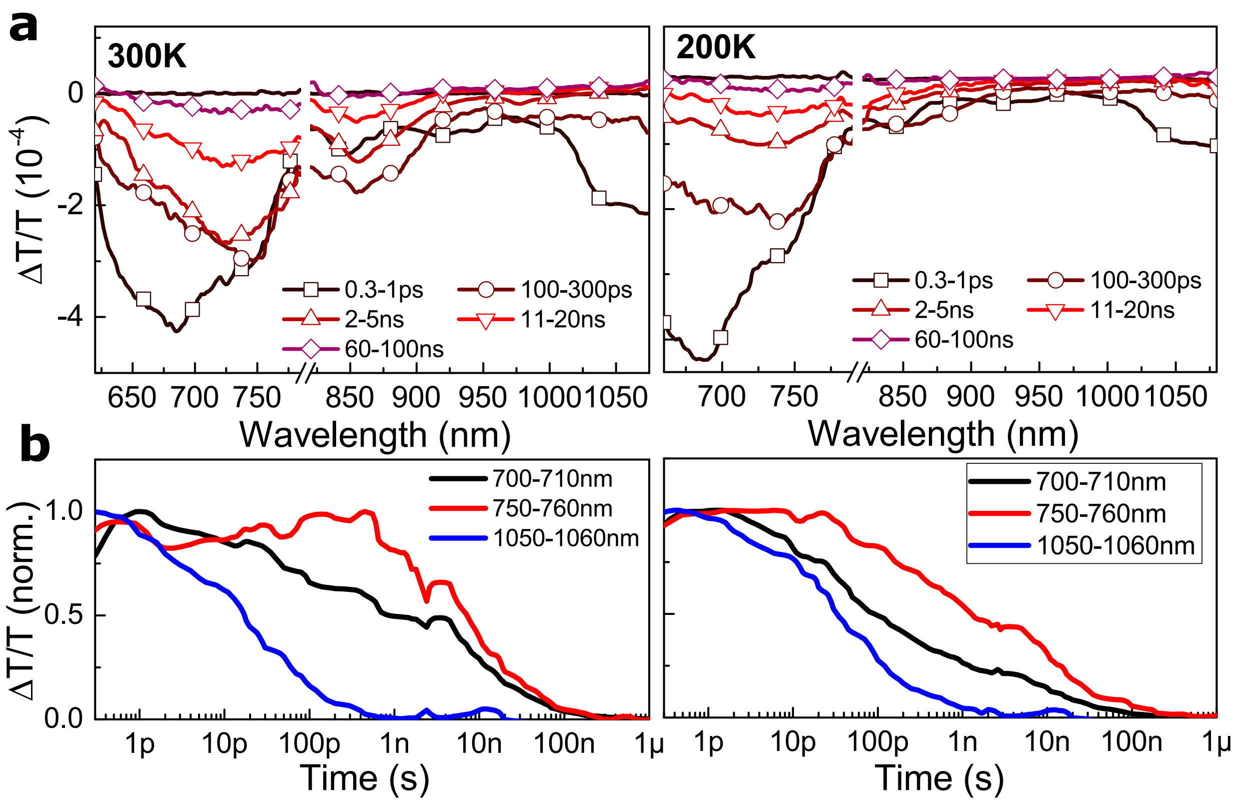

Supplementary Figure 19: **TA of tetracene polycrystalline films.** See discussion in Supplementary Note 3. (a) Transient absorption spectra at 300 K and 200 K. The initial signal peaking at 680 nm is due to singlet excitons [43]. At later times features at 840 nm and 970 nm appear due to triplet exciton absorption. The triplet features are much weaker at 200 K, as seen previously [43]. (b) shows the TA dynamics at selected wavelengths, demonstrating that the singlet dynamics (blue) do not change with temperature.

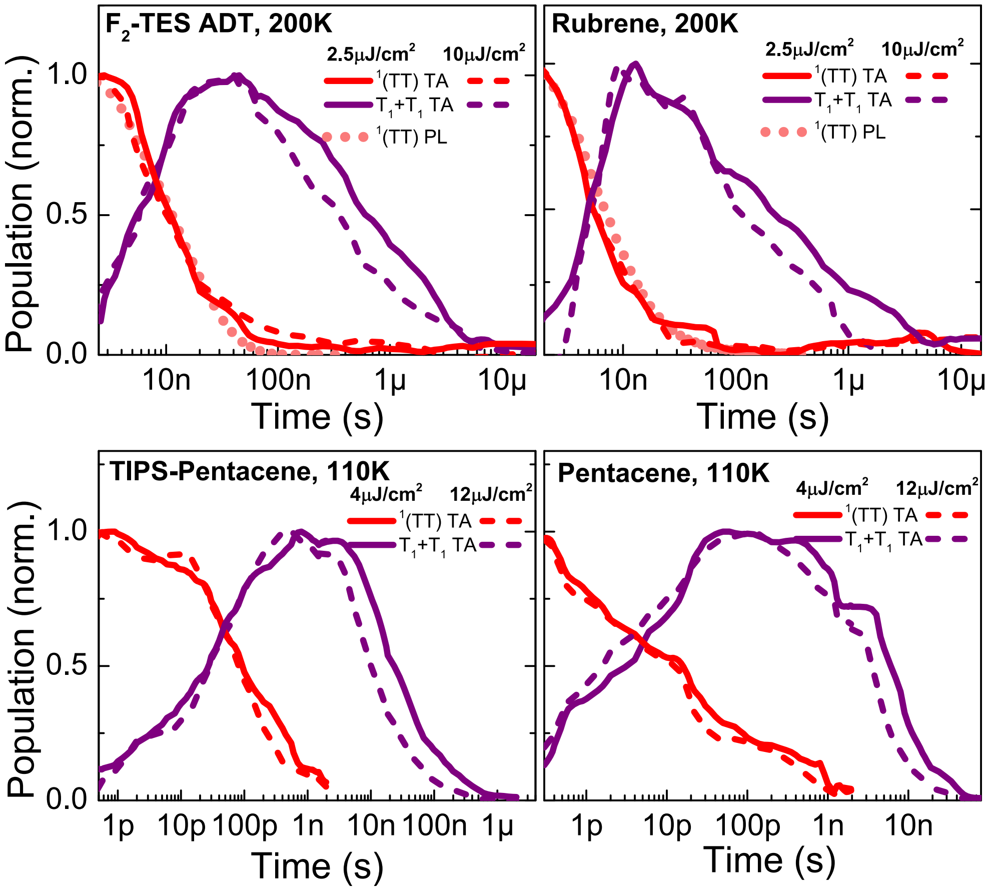

Supplementary Figure 20: **Fluence-dependence of TA dynamics in a variety of materials.** Figure shows dynamics of ^{1}(TT) and free triplets $T_1 + T_1$ in F_2-TES ADT, rubrene, TIPS-pentacene and pentacene extracted from the spectral decomposition methods at two different excitation fluences. For all of the molecules used in this study, except tetracene, for which no determination could be made, we observe that the decay kinetics of ^{1}(TT) (red) are independent of excitation fluence, indicating the geminate nature of the triplet pair in the ^{1}(TT) state. Once the triplet pairs in the ^{1}(TT) state separate, the recombination of these free triplets (purple) exhibits bimolecular triplet-triplet annihilation, where the recombination rate is higher as the excitation density is increased.

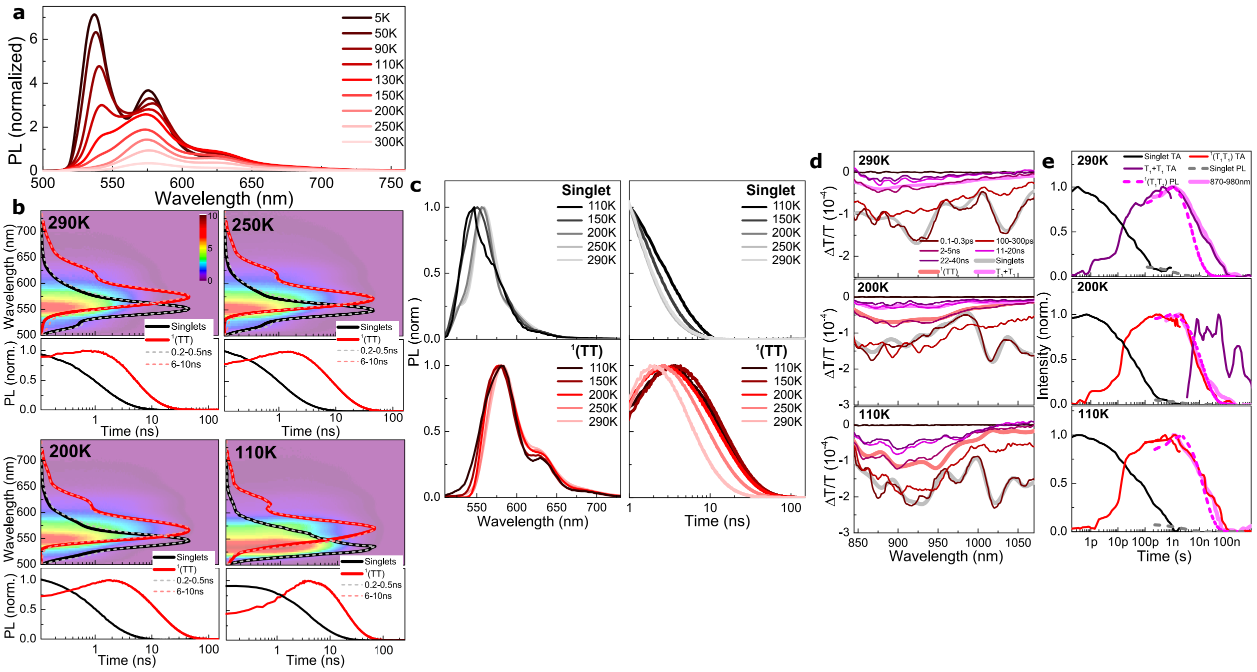

Supplementary Figure 21: **F_2-ADT single crystal PL and TA.** F_2-ADT lacks the solubilising groups of F_2-TES ADT and packs in a herringbone structure. (a) Temperature dependent PL spectra. Despite an obvious blue-shift compared with the F_2-TES ADT films and self-absorption effects, a similar spectral evolution is evident: as the temperature decreases first a red-shifted feature dominates, followed by the super-radiant-like singlet with clearly structured vibronic features. (b) Time-resolved PL maps. Spectral decomposition of the PL maps shows two emitting species whose extracted spectra and lifetimes (solid lines) are plotted with the raw data (dashed lines). Corresponding normalised population kinetics are shown below the maps. (c) Extracted emission spectra and population kinetics of singlet (black) and $^1(\mathrm{TT})$ (red). Interestingly, the singlet lifetime shows a temperature dependence in the single crystal, suggesting singlet exciton fission is activated in the low disorder limit, similar to tetracene [46]. (d) TA spectra. Due to the small crystal size, the pump polarisation was not matched to the crystal direction. Spectral decomposition was performed as for F_2-TES ADT films above. We note that the excited state absorption spectra in our F_2-ADT single crystal are different from the F_2-TES ADT films shown above, which can be attributed to the different molecular packing, as previously observed also in pentacene and its derivatives [38]. At room temperature only two distinct species can be identified, which we attribute to singlets and triplets. At 200 K, three kinetically distinct species are present, which we attribute to singlets, $^1(\mathrm{TT})$ and free triplets. At 110 K, spectral decomposition reveals the presence of only two features: singlets and $^1(\mathrm{TT})$. No free triplets were observed. The corresponding kinetics of each excited state species are shown in (e), together with the kinetics of singlet and $^1(\mathrm{TT})$ emission (dashed).

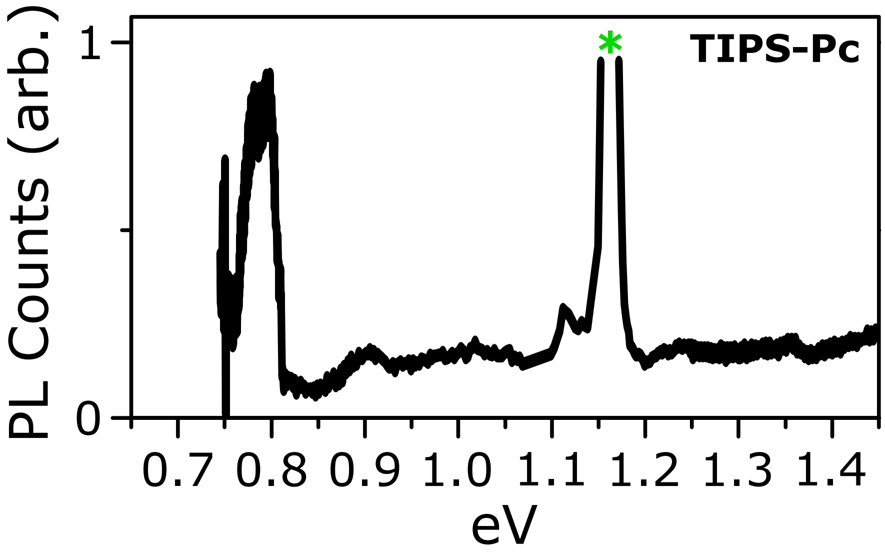

Supplementary Figure 22: **Phosphorescence of a TIPS-pentacene film sensitised with platinum octaethylporphyrin** with excitation at 532 nm. From this the triplet energy in TIPS-pentacene is taken to be 0.79 eV. The green star represents an artefact from the laser at 1.17 eV.

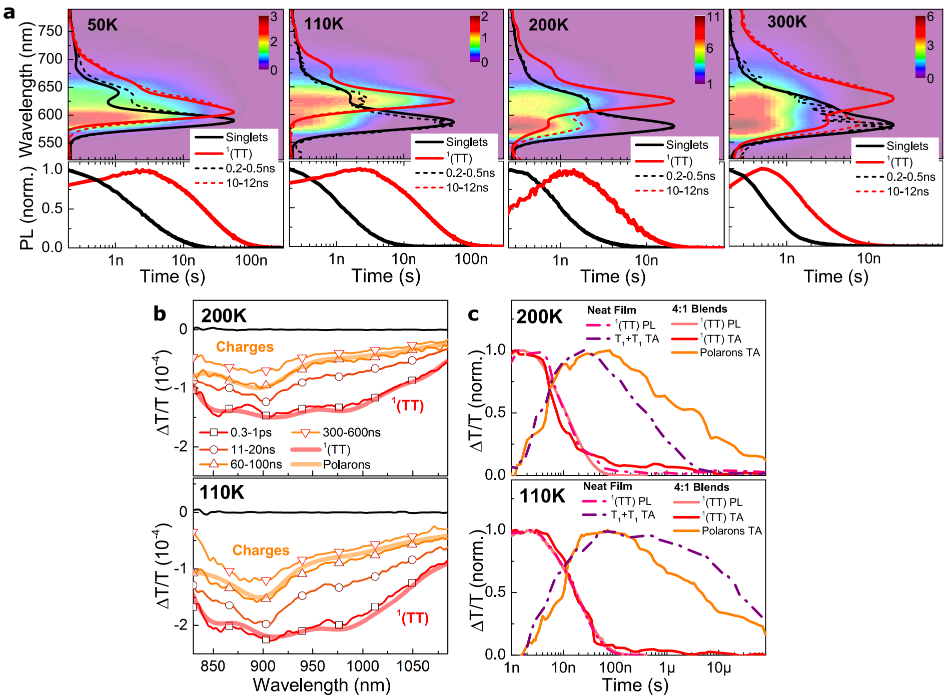

Supplementary Figure 23: **PL and TA of F_2-TES ADT:PC_{71}BM (4:1 molar ratio) blend films.** (a) PL maps taken at 50 K–300 K. As discussed in the main text, emission wavelengths and shapes are similar to those observed in neat films: spectral decomposition analysis yields two kinetically distinct species (singlet and ^{1}(TT)). Raw data (dashed lines) match extracted spectra well. The respective normalised population kinetics are plotted below each PL map. Similar to the 50 K data shown in the main text, at 200 K we see no change in ^{1}(TT) decay in blends compared with neat films.

(b) TA measurements were carried out in the same setup as for neat films. We excited the samples with an excitation pulse centered at 532 nm generated from the second harmonic of Nd:YAG Q-switched laser with a pulse duration of 500 ps. Spectral decomposition analysis suggests the presence of two kinetically distinct species: ^{1}(TT) and charged polarons. Normalised extracted population kinetics shown in (c) are plotted together with the PL and TA kinetics of neat films. 300 K and 50 K data shown in main text.

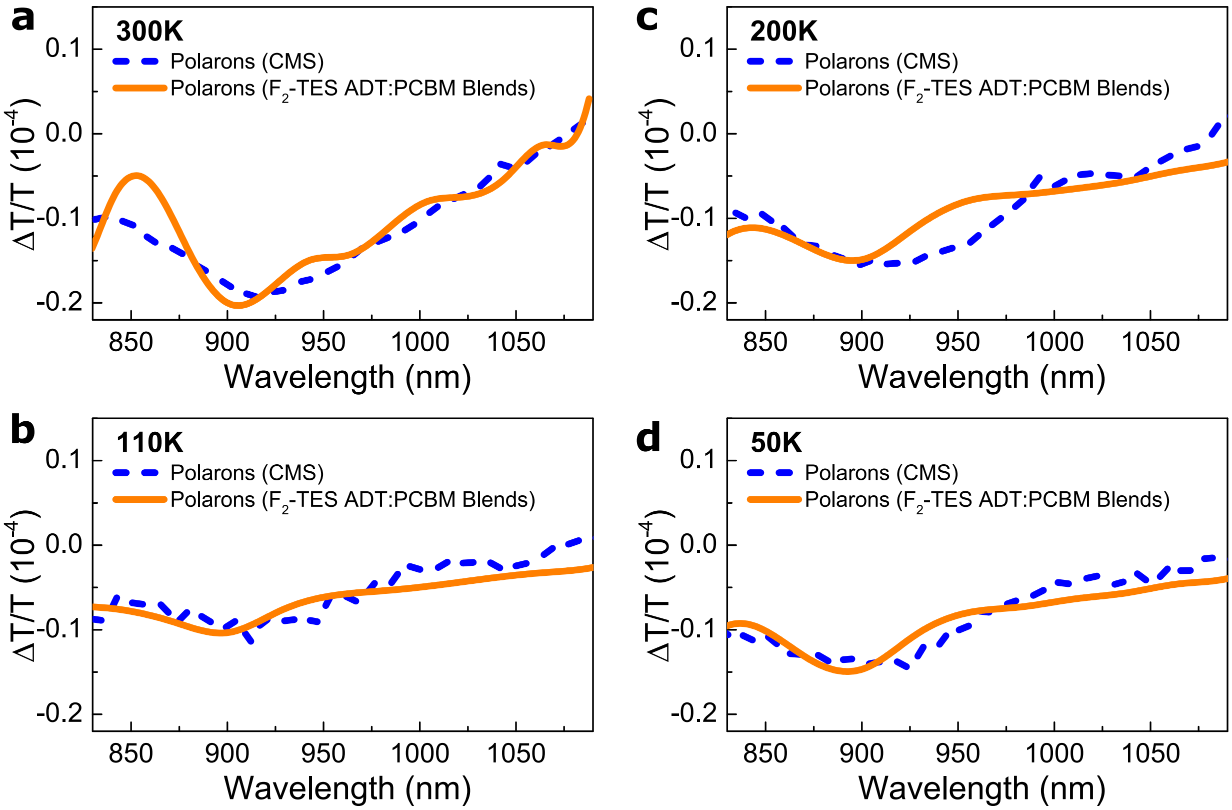

Supplementary Figure 24: **Charge-modulation spectra of F_2-TES ADT.** See also Supplementary Note 5. Measured at (a) 300 K, (b) 200 K, (c) 110 K and (d) 50 K (dashed blue lines). The solid orange lines are spectra for charges extracted by spectral decomposition methods from TA measurements on F_2-TES ADT:PC_{71}BM blends (see supplementary Supplementary Figure 23 and fig. 8 in the main text).

a

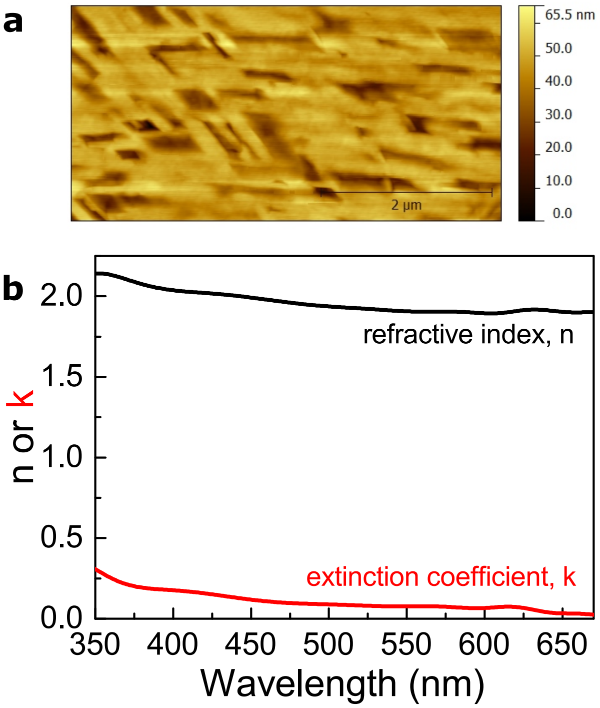

Supplementary Figure 25: **Photovoltaic device characterisation.**(a) AFM image of the F_2-TES ADT:PC_{71}BM layer. (b) Modelled refractive indices (n) and extinction coefficients (k) of films obtained by ellipsometry.

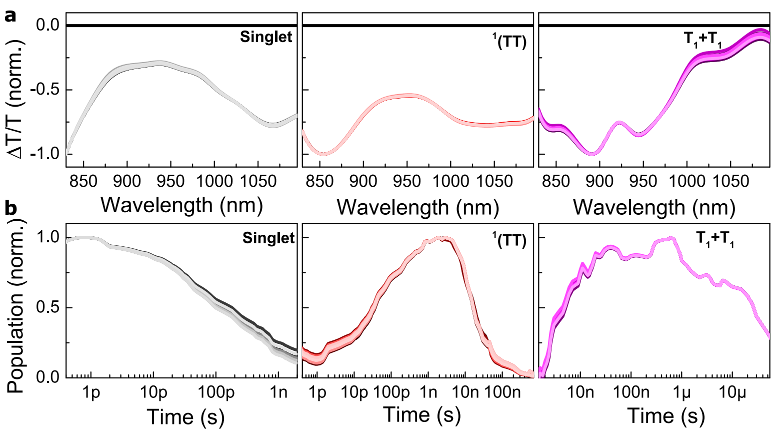

Supplementary Figure 26: **Spectral decomposition via genetic algorithm run multiple times**, giving the resulting (a) spectra and (b) kinetics solutions of singlet, $^1(\mathrm{TT})$ and free triplets populations on a film of F_2-TES ADT at 50 K. The spread of solutions is small, confirming the decomposition method is reliable. See Methods Section in main text for details.

Supplementary Tables

Temperature (K) (K)	Δ T/T$_{CMS}$ (10^{-5})	$\sigma_{pol}{}^{a}$ (10^{-17}cm^2)	n_{ex} Excitation Density (cm^{-2})	Δ A$_p$ (10^{-5})	γ Yield^a
50 K	–	–	7.9×10^{11}	6.02	1.15 (1.26)
110 K	–	–	7.4×10^{11}	5.83	1.19 (1.30)
200 K	–	–	6.7×10^{11}	5.04	1.14 (1.24)
300 K	1.82	6.62 (6.07)	6.0×10^{11}	4.65	1.17 (1.27)

Supplementary Table 1: List of excitation density, cross section, and charge generation yield at different temperatures. Values calculated using Supplementary Figure 5 with ϵ_r=2.4, d=480 nm (C_i=4.4 nFcm^{-2}) and $|\Delta V_g|$=10 V.

^{a} Values in brackets calculated with ϵ_r=2.6, d=480 nm (C_i=4.8 nFcm^{-2}), taken from [79].

Supplementary Notes

Supplementary Note 1 Quantum Beats

Quantum beats in tetracene photoluminescence have been described by Burdett et al. [49]. Here we summarise the origin of the beats.

Singlet fission creates a triplet-pair state in an overall singlet $S = 0$ state. At zero magnetic field, it is convenient to work in the eigenbasis of $\hat{H}_{ZFS}$ which has single triplet eigenstates $|X\rangle$, $|Y\rangle$, $|Z\rangle$ with energies $(D^* - 3E)/3$, $(D^* + 3E^*)/3$, and $-2D^*/3$. Assuming the two triplets are identical, the spin wavefunction for the pure singlet state can be written as a superposition of these single triplet wavefunctions

$$|S\rangle = \frac{1}{\sqrt{3}}(|XX\rangle + |YY\rangle + |ZZ\rangle) \tag{1}$$

For weakly interacting triplet pairs, the zero-field splitting (ZFS) interaction means that $|S\rangle$ is not an energy eigenstate. Instead these are the product states $|XX\rangle$, $|YY\rangle$, $|ZZ\rangle$.

A triplet-pair initially prepared in the state $|\psi(0)\rangle$ = $|S\rangle$ will therefore evolve in time according to the time-dependent Schrödinger equation

$$|\psi(t)\rangle = \frac{1}{\sqrt{3}}(e^{-i\omega_{XX}t}|XX\rangle + e^{-i\omega_{YY}t}|YY\rangle + e^{-i\omega_{ZZ}t}|ZZ\rangle) \qquad (2)$$

where $\hbar\omega_{XX}$ is the energy of the state $|XX\rangle$ and likewise for $|YY\rangle$, $|ZZ\rangle$.

The emission is determined by the overlap of $|\langle S|\psi(t)\rangle|^2$, i.e. the singlet character of the triplet-pair as a function of time

$$|\langle S|\psi(t)\rangle|^2 = 1 + \frac{2}{3}\Big((\cos((\omega_{XX} - \omega_{YY})t) + \cos((\omega_{XX} - \omega_{ZZ})t) + \cos((\omega_{YY} - \omega_{ZZ})t)\Big).$$

The beat frequencies we measure (fig. 3, main text) are 2.9±0.1, 2.0±0.1 and 1.1±0.1 GHz, which compare well with those measured in tetracene (2.99, 1.80, 1.08 GHz) [49]. Using

$$\begin{aligned} |\omega_{XX} - \omega_{YY}| &= 4|E^*| && (3) \\ |\omega_{XX} - \omega_{ZZ}| &= 2|D^* + E^*| && (4) \\ |\omega_{YY} - \omega_{ZZ}| &= 2|D^* - E^*| \end{aligned}$$

we estimate approximate ZFS of $E^*\sim0.72\pm0.03$ GHz and $D^*\sim0.26\pm0.08$ GHz, again comparable with tetracene EPR zero-field splitting parameters in the crystal basis[80] (E^*=0.74 GHz and D^*=0.19 GHz).

This similarity in beat frequencies and corresponding ZFS parameters between F_2-TES ADT and tetracene is puzzling, as F_2-TES ADT contains only one rotationally inequivalent molecule

per unit cell, compared with two in tetracene. In addition, ODMR should provide ZFS parameters which correspond directly to the beat frequencies (i.e. 'crystal-basis' parameters, averaged over the rotationally inequivalent sites in the unit cell), yet the values we obtain (E^*=0.03 GHz and D^*=1.30 GHz) are most comparable to molecular values and different from those we obtain from the beating. We note that our values are presented using two different conventions to ease comparison with literature values: D>E/3 (ODMR) and D<E/3 (quantum beating), which are related by a change of basis. Even accounting for the different bases, the ZFS parameters extracted from ODMR and PL quantum beating differ significantly. It is unclear what gives rise to this discrepancy, namely that in F_2-TES ADT with *one* rotationally inequivalent molecule per unit cell we get beating frequencies similar to tetracene with *two* rotationally inequivalent molecules per unit cell. We propose that the similarity of the beat frequencies could be explained by a transient photo-induced dimerisation altering the intermolecular geometry of F_2-TES ADT during the ^{1}(TT) lifetime to a structure with two rotationally inequivalent molecules per unit cell. The involvement of such symmetry-breaking distortions is consistent with proposed models of vibrationally-mediated singlet fission and our mechanism for ^{1}(TT) emission. A further consideration which may contribute is the relevant timescales of the two measurements ($\leq$ 10 ns for quantum beating, versus μs–ms for ODMR). This makes ODMR more sensitive to longer-lived states, e.g. at trap sites, which could present different ZFS parameters. Finally, we note that interaction between the triplets within the pair could modify the beat frequencies [49]. We have modelled this by including a term in the Hamiltonian $H_J = J\mathbf{S_1} \cdot \mathbf{S_2}$ where $\mathbf{S_1}, \mathbf{S_2}$ are the spin operators for the two triplets. The beat frequencies as a function of the interaction strength J are shown in 7. The frequencies increase

with increasing interaction strength but do not quantitatively match our experiments. This mismatch indicates either that the surprising discrepancy between observed beat frequencies and ZFS parameters arises from different factors (i.e. transient photodimerisation) or that a more precise anisotropic model of the spin interaction is needed.

Supplementary Note 2 Optically detected magnetic resonance (ODMR)

Supplementary Figure 6 shows the changes in photoluminescence due to magnetic resonance as a function of magnetic field at 45 K, normalised by the steady-state PL intensity, along with a fit to a density-matrix simulation (similar to as described in [81]) which yields zero-field splitting (ZFS) parameters (in frequency units) of $D^* \sim 1.30\,\mathrm{GHz}$, and $E^* \sim 0.03\,\mathrm{GHz}$, following the standard ODMR convention of D>E/3. These parameters are similar to typical molecular ZFS parameters in acenes[80,82]. The characteristic triplet powder pattern identifies the species involved as triplet excitons, and the superlinear intensity dependence of the ODMR signal (Supplementary Figure 6(b)) indicates that the ODMR signal arises from a bimolecular recombination process which we assign to triplet-triplet annihilation (TTA). Magnetic resonance enhances the number of bimolecular TTA events which can give rise to an emissive singlet exciton resulting in an increase in emission under magnetic resonance [83]. The microwave chopping frequency dependence of the ODMR signal is shown in Supplementary Figure 6c along with a fit to a single exponential decay time τ i.e. $|\Delta PL(f_M)/PL| \propto 1/\sqrt{1+(2\pi f_M \tau)^2}$ which yields $\tau = 118\,\mu\mathrm{s}$, a lifetime longer than the primary signals we measure using transient optical spectroscopy, consistent with a sub-

population of trapped triplets.

Supplementary Note 3 Spectroscopy of Tetracene vs F2-TES ADT

Temperature-dependent transient PL Tetracene is one of the best-known materials for singlet exciton fission applications and its emissive properties have been very well studied in recent years [34,41–43,46,49,62,74]. In 1975, Müller and Bässler showed, using temperature-dependent PL measurements on polycrystalline tetracene films [41], that as the films are cooled, a new red-shifted feature (∼570 nm), dominates the spectrum. Other work has confirmed these early findings, an example from Tayebjee et al. [34] is reproduced in Supplementary Figure 8 which also demonstrates (Supplementary Figure 8 (b)) that the 570 nm feature and its vibronic replica are long-lived compared with the prompt singlet emission [34,42].

This spectral behaviour and shape are strikingly similar to that of F_2-TES ADT in fig. 1 of the main text, and we therefore assign the prominent peak at 570 nm to the 0–1 replica of $^1(\mathrm{TT})$ emission. The assignment is confirmed using the Herzberg-Teller model to extract the $^1(\mathrm{TT})$ energy from the PL spectra, Supplementary fig. 1. Assuming the 570 nm feature is the 0–1 component and the effective vibronic energy is 0.15 eV, we estimate the tetracene $^1(\mathrm{TT})$ energy to be 2.32 eV. Comparing this with the free triplet energy in fig. 4 in the main text shows that it follows the trend, as expected.

Supplementary Figure 9a shows the emission spectrum as a function of temperature for a

tetracene thin film prepared by us. While the overall behaviour of the singlet and ^{1}(TT) features are similar to previous reports [34,42], we attribute the increased intensity in the 600–700 nm spectral region, also seen in Ref. [43], to emission from an overlapping excimer state [46]. We prepared several films in order to reduce the presence of this feature, but it was always present to some degree. The presence of this excimer state makes analysis of the transient PL non-trivial and rather than perform a full spectral decomposition on these data, we instead focus on the dynamics at the peak intensity of the singlet 0–0 feature at $\sim$530 nm, and the red-shifted feature in a spectral range where the prompt singlet emission is only weak ($\sim$650 nm). Both dynamics decay non-exponentially suggesting the presence of at least two emissive populations in each spectral range. Interestingly, although intensity is very weak after 8 ns, spectra at this time are dominated by the 'excimer' emission.

Quantum Beating We described quantum beating in F_2-TES ADT in Supplementary Note 1. The beats are present due to the spin entangled nature of ^{1}(TT) and decay over time. We note that quantum beating similar to that shown in fig. 3 in the main text has been measured in tetracene by several groups [49,50,84,85] and we discuss these measurements in comparison with F_2-TES ADT in Supplementary Note 1. The decay of the beats matches the dynamics of the red-shifted feature in the PL spectra at 650 nm, as shown by the solid red line in fig. 3 in the main text and confirms our assignment that, as discussed above, the red-shifted emission can be attributed to ^{1}(TT) emission.

Temperature-dependent transient absorption The transient absorption spectra of tetracene as a function of temperature are notoriously difficult to measure [43,62]. As the sample cools it scatters, significantly decreasing the S/N ratio [43]. At the same time, the unusually low excited-state absorption cross-sections in our probe region[43,59], which tend to change with morphology [60,61], coupled with the requirement for low excitation density to avoid exciton-exciton annihilation[62], make this experiment especially tricky. Previous low temperature TA measurements of tetracene required probing at single wavelengths at the laser fundamental (800 nm) to provide enough S/N to measure at low fluences below room temperature. We have therefore restricted our measurements to room temperature and 200 K and probed in a broader spectral range (680-1100nm). Supplementary Figure 19 shows TA spectra of tetracene films at 200 K and 300 K.

We find clear triplet features in the 300 K spectra with distinct peaks at 840 nm and 970 nm, similar to TIPS-tetracene [22]. However, similar to F_2-TES ADT at this temperature (fig. 5 in the main text and fig. 13), we find no distinct transient absorption signature of $^1(\mathrm{TT})$ due to an effective thermal equilibrium between $^1(\mathrm{TT})$ and T_1+T_1. Although the singlet fission kinetics remain identical at 200 K, the triplet excited-state absorption features are much weaker precluding any identification of distinct excited-states. An analogous result was found in an earlier study of polycrystalline tetracene films [43]. We speculate the drop in oscillator strength from 300–200 K is due to a subtle morphological change, in line with the morphology-dependent $T_1 \rightarrow T_2$ oscillator strength in these materials [43,59–61].

Conclusion We conclude by pointing out that the PL behaviour of polycrystalline tetracene [34,42,43] and F_2-TES ADT thin films are almost identical, showing similar $^1(\mathrm{TT})$ behaviour as a function of temperature, PL lifetimes and quantum beating. The similarity of these features suggest that the photophysics is similar in both material sets. It is unfortunate that tetracene thin films are difficult to measure using transient absorption spectroscopy at low temperature, making assignment of the $^1(\mathrm{TT})$ excited-state absorption features almost impossible. For this reason we focus in our TA studies on materials where the triplet TA signatures are easier to observe such as F_2-TES ADT, rubrene and pentacene.

Supplementary Note 4 1(TT) in Pentacene Derivatives

It is now very well established that both pentacene and TIPS-pentacene undergo singlet fission to generate free triplets. The TA spectrum at long times (1 ps-1 ns depending on temperature) is therefore assigned to free triplets in the literature. The spectral evolution that occurs prior to the 'final' relaxed spectrum (see for example supplementary figs. 16c and 18c) has only recently been addressed in the literature[78]. Although published data demonstrates similar evolution, it only becomes clearly apparent at low temperatures: the spectral evolution slows down as the temperature is lowered. This spectral evolution could be due to triplet exciton migration, however we attribute it to evolution between different excited states for the following reasons:

1. we used identical spectral decomposition methods for the pentacenes as for the other ma-

terials and these yield very similar dynamic results. Comparison with the other materials (F_2-TES ADT, rubrene, TIPS-tetracene [21,26]), allows us to assign the intermediate to ^{1}(TT) also in pentacenes.

2. in TA measured below 10 K (in F_2-TES ADT, both pentacenes), we see a secondary growth of the NIR signal, peaking between 10-100ns (figs. 13b; 16b; 17b). Such a late-time growth in signal is unlikely to be due to triplet migration. Instead we suggest it demonstrates the presence of two independent species absorbing in this spectral region. The clear separation between decay of the first species and rise of the second enables us to separate the spectra of the two species below 10 K. The extracted spectra at these temperatures match those extracted at higher temperatures, confirming our assignment of an intermediate species in TIPS-pentacene, pentacene and F_2-TES ADT. We assign this intermediate species to ^{1}(TT) in F_2-TES ADT (main text) and the uncanny similarity in dynamics between F_2-TES ADT and the pentacenes likewise allows us to assign the intermediate in pentacenes to bound, entangled ^{1}(TT).

Supplementary Note 5 Charge generation yield estimation

To determine the optical cross section of the hole polaron in F_2-TES ADT, we performed charge modulation spectroscopy. The CMS experiments have been described in detail elsewhere[86]. Briefly, a top-gate, bottom-contact transistor was fabricated on $2 \times 2\,cm^2$, low alkali content borosilicate glass (Corning 1737F, 0.7 mm thick), with cytop as dielectric layer (dielectric constant ϵ_r=2.4,

thickness d=480 nm). As an organic material coating favours the wetting of organic semiconductors, a 50 nm-thick precursor of polyimide (PI-2525, HD MicroSystems) was spin-coated onto the cleaned glass substrates and cross-linked under 300°C prior to spin-coating of the active layer. For CMS measurements, the device was mounted in a cryostat with dynamic helium gas flow and illuminated with monochromatic light. A constant d.c. voltage of 45 V was applied to the gate in order to accumulate the charge layer near the dielectric. An a.c voltage $\pm$ 5 V was applied on top of a d.c voltage on the gate. The frequency of modulation was 27 Hz. In this way, the amount of charge stored in the device could be modulated, resulting in a corresponding modulation of the transmittance of the incident monochromatic light (0.5 to 3.0 eV). This was detected using lock-in techniques. The resulting spectra can be conceptually imagined as a difference spectrum between the device biased at $V+\delta V_{ac}$ and $V-\delta V_{ac}$. Supplementary Figure 24 shows the CMS spectra of a F_2-TES ADT film at different temperatures. We observe broad absorption features of the charge in the channel at wavelengths between 830 nm and 1090 nm. The spectral shape is consistent with the charge spectra extracted from TA measurements of F_2-TES ADT:$PC_{71}BM$ blends by spectral decomposition methods (see main text).

The modulated charge density can be calculated from a capacitance equation. Here we use the simplifying assumption that the transistor has low contact resistance, high mobility and operates in full accumulation. The corresponding cross section of charges σ_{pol} can be estimated based on the following equation[86]:

$$-\Delta \mathrm{T}/\mathrm{T}_{\mathrm{CMS}} = \sigma_{\mathrm{pol}} \times \mathrm{n_c} = \frac{\sigma_{\mathrm{pol}}\mathrm{C_i}}{\mathrm{e}}|\Delta \mathrm{V_g}| \quad (5)$$

where $\Delta T/T_{CMS}$ is the change of transmission of charges measured in the CMS (Supplementary Figure 24), n_{c} is the charge density per unit area, which can be calculated based on a parallel plate capacitance model, *e* is the electron charge, C_i is the capacitance per unit area and $|\Delta V_g|$ is the modulated gate voltage (10 V). With a dielectric constant of 2.4 and a dielectric thickness of $d = 480$ nm, the capacitance per unit area is C_i=4.4 nFcm^{-2}. C_i is known to vary depending on thickness of the dielectric layer, for example literature values for cytop [79] give 28 nFcm^{-2}, 14 nFcm^{-2} and 5.2 nFcm^{-2} for 120-nm-, 250-nm- and 450-nm-thick films, respectively, with ϵ_r varying from 3.9 to 2.6. Thin films show higher effective dielectric constants than thick films due to screening from the metal surfaces. We therefore take our value of C_i=4.4 nFcm^{-2} (ϵ_r=2.4) to be a lower limit for a 480 nm- thick cytop film.

We calculated the optical cross-section of charges based on the CMS data at room temperature and assume the cross-section is independent of temperature. At lower temperatures, due to increased contact resistance and reduced mobility, the calculation of the cross-section is more error-prone. The change of absorption due to the presence of charges in the F_2-TES ADT:PC_{71}BM blends ΔA_p is extracted from the spectral decomposition analysis. We take the maximum of ΔA_p to calculate the maximal charge yield using $-\Delta \mathrm{A_p} = \sigma_{\mathrm{pol}} \times \mathrm{n_d}$ and the resulting charge yield is then estimated using $\gamma = \frac{\mathrm{n_{ex}}}{\mathrm{n_d}}$. All values used for the calculation are shown in Supplementary Table 1 and the charge yields are also shown in the main text.

Supplementary References

74. Burdett, J. J., Müller, A. M., Gosztola, D. & Bardeen, C. J. Excited state dynamics in solid and monomeric tetracene: The roles of superradiance and exciton fission. *J. Chem. Phys.* **133**, 1–12 (2010).

75. Lehnherr, D. *et al.* Isomerically Pure syn-Anthradithiophenes: Synthesis, Properties, and FET Performance. *Org. Lett.* **14**, 3660–3663 (2012).

76. Wen, X., Yu, P., Yuan, C.-T., Ma, X. & Tang, J. Singlet and Triplet Carrier Dynamics in Rubrene Single Crystal. *J. Phys. Chem. C* **117**, 17741–17747 (2012).

77. Poletayev, A. D. *et al.* Triplet Dynamics in Pentacene Crystals: Applications to Fission-Sensitized Photovoltaics. *Adv. Mater.* **26**, 919–924 (2014).

78. Pensack, R. D. *et al.* Observation of Two Triplet-Pair Intermediates in Singlet Exciton Fission. *J. Phys. Chem. Lett.* **7**, 2370–2375 (2016).

79. Sakanoue, T. & Sirringhaus, H. Band-like temperature dependence of mobility in a solution-processed organic semiconductor. *Nat. Mater.* **9**, 736–740 (2010).

80. Yarmus, L., Rosenthal, J. & Chopp, M. EPR of triplet excitions in tetracene crystals: spin polarization and the role of singlet exciton fission. *Chem. Phys. Lett.* **16**, 477–481 (1972).

81. Bayliss, S. L. *et al.* Geminate and Nongeminate Recombination of Triplet Excitons Formed by Singlet Fission. *Phys. Rev. Lett.* **112**, 238701 (2014).

82. Von Schuetz, J., Gueckel, F., Steudle, W. & Wolf, H. C. Phosphorescence- and delayed-fluorescence-detected magnetic resonance at zero-field on mobile and localized triplet states in anthracene. *Chem. Phys.* **53**, 365–372 (1980).

83. Benfredj, A., Henia, F., Hachani, L., Romdhane, S. & Bouchriha, H. Theoretical and experimental study of dynamic triplet-triplet annihilation in an organic one-dimensional motion system. *Phys. Rev. B* **71**, 75205 (2005).

84. Chabr, M. & Wild, U. P. Quantum Beats of Prompt Fluorescence in Tetracene Crystals. *Chemical Physics* **57**, 425–430 (1981).

85. Fünfschilling, J. Quantum Beats in the Fluorescence Decay of Tetracene Crystals. *Le J. Phys. Colloq.* **46**, 377–380 (1985).

86. Brown, P. J., Sirringhaus, H., Harrison, M., Shkunov, M. & Friend, R. H. Optical Spectroscopy of Field-induced Charge in Self-organized High Mobility Poly(3-hexylthiophene). *Phys. Rev. B* **63**, 125204–125215 (2001).
